# S100A9 as a Candidate Molecular Bridge in Hepato–Ocular Crosstalk

**DOI:** 10.3390/biology15151306

**Published:** 2026-08-05

**Authors:** Peng Wang, Yamei Li, Bohou Xia, Yan Lin, Qinhui Tuo, Limei Lin, Qiuxian Peng

**Affiliations:** Key Laboratory for Quality Evaluation of Bulk Herbs of Hunan Province, School of Pharmacy, Hunan University of Chinese Medicine, Changsha 410208, China

**Keywords:** S100A9, hepato–ocular crosstalk, alarmin, TLR4-associated signaling, diabetic retinopathy, translational implications

## Abstract

Increased S100 calcium-binding protein A9 (S100A9)-related signals have been reported in selected hepatic and ocular inflammatory settings. This structured narrative review evaluates S100A9-related species as candidate participants in hepato–ocular crosstalk. Across human, ocular fluid, animal, and cellular studies, the available findings provide context-specific support for disease-associated hepatic expression and ocular responsiveness, with stronger evidence for selected local S100A9–Toll-like receptor 4 (TLR4)-associated effects than for S100A9-specific receptor for advanced glycation end products (RAGE) signaling. Clinical associations involving metabolic dysfunction-associated steatotic liver disease, diabetic retinopathy, chronic liver disease, dry eye disease, and uveitis are heterogeneous and confounded. Interpretation is further limited by the non-equivalence of S100A9, S100A8/A9, calprotectin, and higher-order complexes. Current evidence, therefore, suggests that S100A9-related species may serve as exploratory indicators of inflammatory activity or contribute to local inflammatory amplification in selected settings, rather than acting as established liver-derived causal signals. Future studies should prioritize analyte-specific measurement, source tracing, and selective perturbation. S100A9 is best regarded as a testable candidate node within a broader metabolic–inflammatory network.

## Simple Summary

Liver and eye disorders can coexist; however, it remains uncertain as to whether specific inflammatory mediators connect them. S100A9 is an inflammation-associated protein expressed mainly by myeloid cells; increased S100A9-related signals have been reported in selected hepatic and ocular conditions. This review examines evidence involving the retina, ocular surface, and uvea. Current evidence supports context-dependent local ocular responsiveness, whereas a direct liver-to-eye route has not been demonstrated. S100A9-related species may reflect systemic inflammation or contribute to local inflammatory amplification in selected ocular models; however, their tissue source and molecular form remain unresolved. Future studies should distinguish S100A9 from S100A8/A9 complexes, identify their cellular and organ sources, and directly test systemic transport and ocular target engagement before clinical applications are considered.

## 1. Introduction

Physiological and clinical associations between the liver and eye have been recognized in selected contexts, including systemic vitamin A homeostasis [1]. Within this review, “hepato–ocular crosstalk” refers to potential interactions through circulating factors, immune cells, extracellular vesicles, or shared systemic conditions, without presuming a unidirectional liver-to-eye pathway. Hereditary transthyretin amyloidosis provides an instructive comparator: although the liver is a major source of circulating variant transthyretin, ocular manifestations may persist or emerge after liver transplantation, consistent with continued transthyretin production within the eye [2,3]. This example illustrates the need to distinguish systemic source contributions from local ocular production.

Clinical liver–eye associations remain heterogeneous. Metabolic dysfunction-associated steatotic liver disease (MASLD) has been associated with proliferative or treated diabetic retinopathy (DR) in some adjusted observational studies, whereas other cohorts and meta-analyses have reported null, inverse, or disease stage-dependent relationships [4,5,6,7]. Ocular surface abnormalities and dry eye disease (DED) have, likewise, been described in selected liver disorders [8]. Interpretation is complicated by diabetes severity and cardiometabolic comorbidity [4,5,6,7], heterogeneity among liver-disease categories [8], and socioeconomic differences [9]. A local ocular contribution is also plausible because S100A9-related changes have been reported in retinal, ocular surface, and anterior uveitis models [10,11,12,13]. These observations support hypothesis generation but do not establish inter-organ signaling.

Current MASLD, metabolic dysfunction-associated steatohepatitis (MASH), and metabolic dysfunction and alcohol-associated liver disease (MetALD) terminology follows the 2023 multi-society consensus [14]. Legacy terms are retained only when describing original studies, and other liver diseases are treated as distinct clinical or experimental contexts.

S100 calcium-binding protein A9 (S100A9) is a myeloid-enriched damage-associated molecular pattern involved in innate immune signaling [15,16,17,18]. Disease-associated S100A9- or S100A8/A9-related signals have been reported predominantly in hepatic myeloid populations across selected steatotic, fibrotic, septic, toxic, infectious, and ischemia–reperfusion settings [19,20,21,22,23,24,25,26,27], including S100A9-high macrophage populations in human MASLD/MASH single-cell datasets [23]. These studies support disease-associated hepatic expression but do not resolve the quantitative contribution of the liver to circulating S100A9-related material. Interpretation is further complicated by the non-equivalence of S100A9 transcripts or intracellular protein, soluble extracellular S100A9, S100A8/A9 heterocomplexes, higher-order assemblies, phosphorylated S100A9, and extracellular vesicle-associated material [15,18,28,29,30,31]. The assay design and biological matrix may further affect interpretation [32].

Selected ocular models provide model-specific support for S100A9–Toll-like receptor 4 (TLR4)-associated responses in experimental DR and DED [10,11], while experimental deletion studies indicate cell type-dependent TLR4 functions in the diabetic retina [33]. By contrast, an ocular S100A9-specific receptor for advanced glycation end products (RAGE) engagement has not been directly demonstrated; available RAGE studies using advanced glycation products, matrix-associated stimuli, or HMGB1 are considered to have an indirect biological context [34,35,36]. Because these receptors and their downstream pathways respond to multiple metabolic and inflammatory stimuli, pathway activation alone cannot identify S100A9 as the initiating ligand [33,34,35,36,37]. Accordingly, this structured narrative and hypothesis-generating review evaluates disease-associated hepatic expression and potential systemic availability of S100A9-related species, context-dependent ocular responsiveness, and compartment-specific evidence in DR, DED, and selected forms of uveitis. S100A9 is assessed as a testable candidate node rather than as an established hepato–ocular causal bridge.

## 2. Review Approach, Evidence Identification, and Claim-Level Appraisal

This article was developed as a structured narrative and hypothesis-generating review integrating clinical, ocular fluid, animal, cellular, and molecular evidence relevant to the proposed S100A9-related hepato–ocular framework. Its organization and appraisal were informed by the Scale for the Assessment of Narrative Review Articles (SANRA) [38]. Because it was not designed as a systematic or scoping review, exhaustive retrieval, quantitative synthesis, formal study-level risk-of-bias assessment, and Grading of Recommendations Assessment, Development, and Evaluation (GRADE)-based certainty evaluation were not undertaken.

Targeted searches of PubMed/MEDLINE, Embase, Web of Science Core Collection, and Scopus covered database inception to 1 May 2026. Search terms combined S100A9 or S100A8/A9 with hepatic diseases, ocular tissues or disorders, receptor signaling, circulating or ocular fluid biomarkers, and experimental interventions; legacy NAFLD and NASH terms were retained. Database-specific search strategies are provided in Supplementary Table S1. Additional information on evidence selection, claim-level appraisal, disagreement resolution, and reporting conventions is provided in Supplementary Text S1.

## 3. Hepatic Sources and Potential Systemic Availability of S100A9-Related Molecular Species

### 3.1. Hepatic Cellular Sources and Disease-Associated Expression

Selected forms of metabolic liver injury and fibrosis are accompanied by hepatic myeloid remodeling [23,39]. In selected steatotic, fibrotic, septic, toxic, infectious, and ischemia–reperfusion settings, S100A9- or S100A8/A9-related signals have been reported, mainly in Kupffer cells, recruited macrophages, and neutrophils [19,20,21,22,23,24,25,26,27]. A human single-cell study identified S100A9-high hepatic myeloid populations, including TREM2^+^S100A9^+^ macrophages, across the MASLD/MASH stages examined [23]. An experimental fatty liver model further implicated neutrophil-derived S100A8/A9 in tissue injury and myofibroblast migration [21]. By contrast, non-malignant hepatocytes were not identified as a major S100A9-expressing population in the cited human single-cell dataset, leaving hepatocyte involvement provisional [23]. These findings provide context-specific support for disease-associated hepatic myeloid expression and local inflammatory involvement but do not quantify the hepatic contribution to circulating S100A9-related species.

### 3.2. Molecular Forms, Release, and Intrahepatic Amplification

Extracellular activity may vary according to molecular form. S100A8 and S100A9 preferentially form heterodimers and can assemble into calcium-dependent heterotetramers; isolated S100A9, total S100A8/A9 immunoreactivity, and unresolved oligomeric mixtures should, therefore, not be treated as interchangeable analytes or biological entities [15,18,28,29]. Thr113-phosphorylated S100A9 has been reported to be released from activated neutrophils [30]. Evidence concerning gasdermin D-associated release, neutrophil extracellular trap-associated release, and extracellular vesicle carriage derives predominantly from non-hepatic systems and is, therefore, treated as an indirect biological context [31,40,41,42,43].

**Figure 1:**
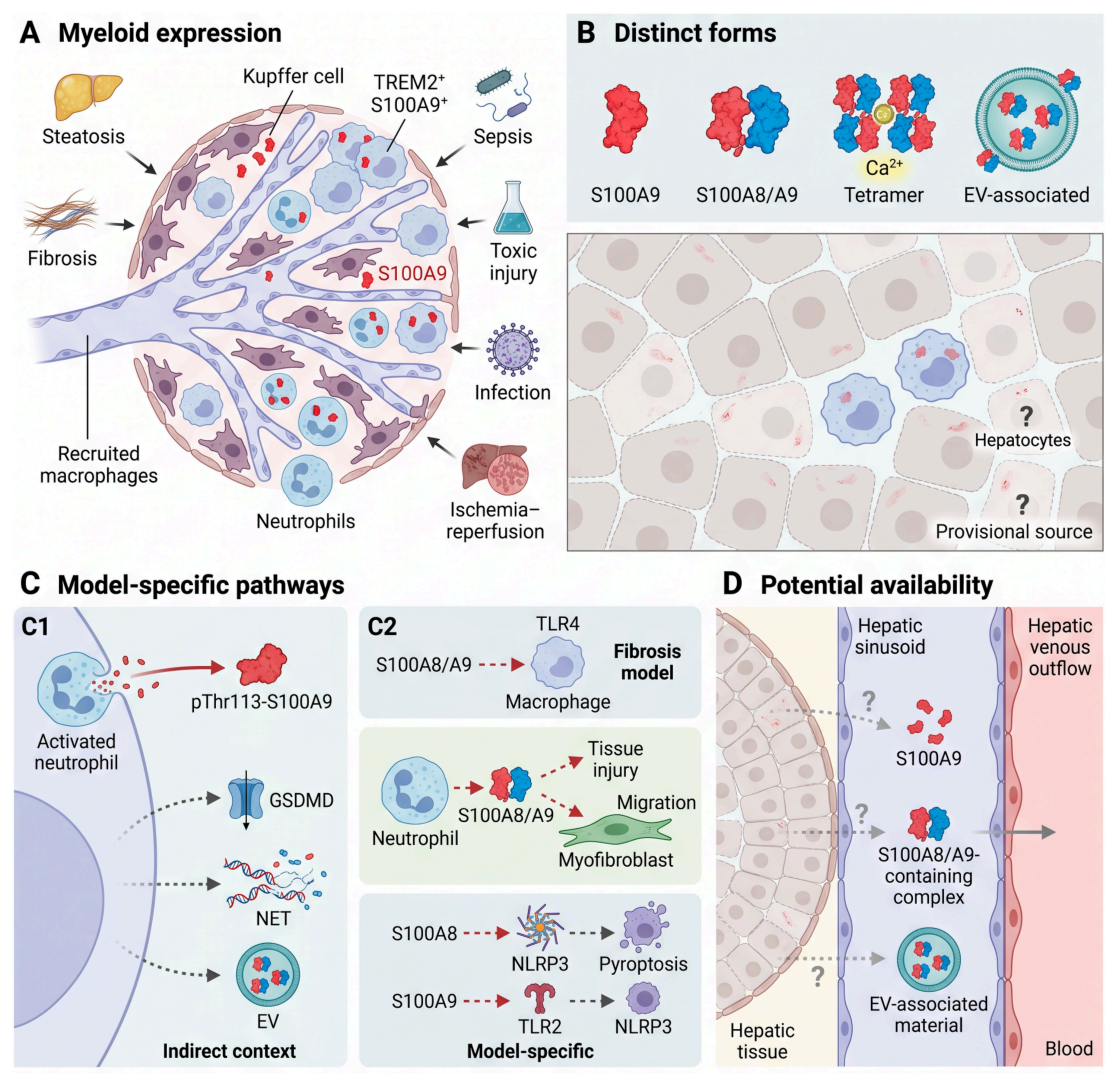
Hepatic sources, molecular forms, release context, and potential systemic availability of S100A9-related molecular species. Disease-associated hepatic expression is supported mainly in Kupffer cells, recruited macrophages, and neutrophils, whereas the contribution of non-malignant hepatocytes remains provisional. Isolated S100A9, S100A8/A9 heterodimers, calcium-dependent heterotetramers, and extracellular vesicle-associated material are shown separately because they should not be assumed to be biologically or analytically interchangeable. Release mechanisms and intrahepatic signaling are presented according to their specific experimental contexts rather than as a unified pathway. Potential entry into the circulation remains unresolved and does not establish the liver as the dominant source of circulating S100A9-related analytes. Solid arrows indicate context-specific direct support for the depicted hepatic claim component; dashed arrows indicate animal model-specific evidence; dotted grey arrows indicate indirect biological context; and grey arrows marked with a question mark indicate unresolved or hypothetical relationships. Created in BioRender. Wang, P. (2026) https://BioRender.com/4bitthv (accessed on 30 June 2026).

Within specific liver models, different S100A9-related molecular species have been implicated in distinct signaling or injury-associated processes: S100A8/A9 in macrophage TLR4-associated signaling during fibrosis [19], neutrophil-derived S100A8/A9 in fatty liver injury and myofibroblast migration [21], S100A8 in NLRP3-dependent macrophage pyroptosis [44], and S100A9 in TLR2–NLRP3-associated signaling during fatty liver ischemia–reperfusion injury [26]. Collectively, these findings are consistent with model-specific intrahepatic inflammatory amplification but do not support a single unified S100A9 pathway (Figure 1).

### 3.3. Systemic Availability, Source Attribution, and Biomarker Interpretation

Sinusoidal fenestration provides an anatomically permissive interface between hepatic tissue and blood; however, this anatomical relationship alone does not demonstrate hepatic release or quantify source contribution [39]. Circulating measurements may capture soluble S100A9, S100A8/A9 complexes, or extracellular vesicle-associated material; current vesicle evidence does not establish hepatic origin or ocular delivery [15,28,29,31].

In cirrhosis, much of the available literature concerns fecal or ascitic calprotectin rather than circulating isolated S100A9 [45]. In type 2 diabetes, circulating S100A8 and S100A9 were cross-sectionally associated with DR severity; however, these findings do not establish hepatic mediation [46]. Serum and plasma measurements also require separate pre-analytical interpretation [32]. Circulating S100A9-related analytes should, therefore, be regarded as exploratory biomarker candidates pending analyte-specific, matrix-specific, and incremental clinical validation.

## 4. Ocular Receptor Competence

**Figure 2:**
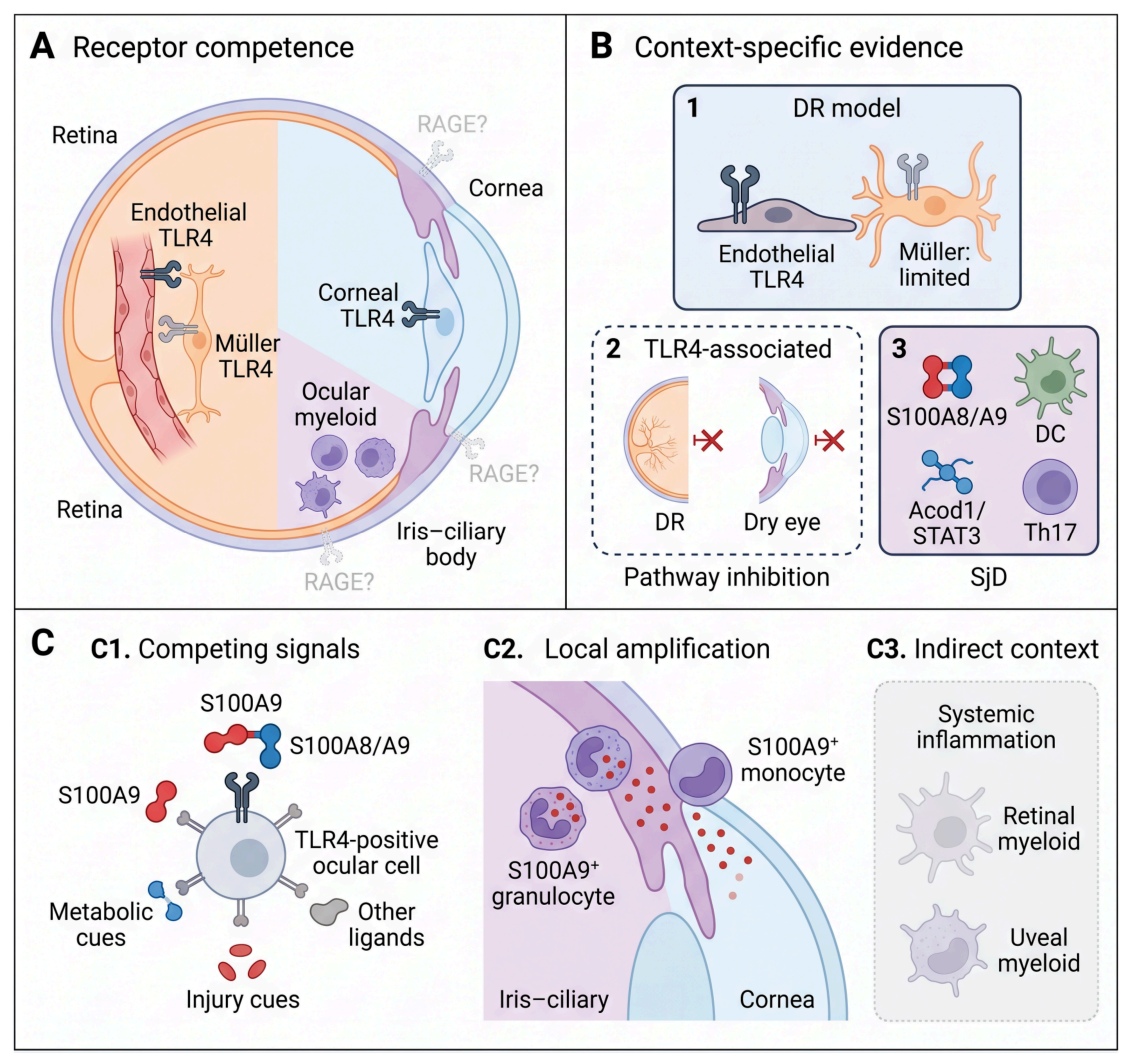
Ocular receptor competence, context-specific evidence, and potential local ocular inflammatory amplification involving S100A9-related species. Context-specific TLR4 competence is supported in retinal endothelial cells, Müller cells, and corneal epithelial cells, with stronger functional evidence for retinal endothelial cells than for Müller cells in the cited diabetic retina deletion model. RAGE is depicted only as a limited and unresolved candidate receptor because direct ocular S100A9–RAGE engagement has not been demonstrated. Separate modules summarize model-associated S100A9/TLR4-related findings in DR and DED, the distinct S100A8/A9–Acod1/STAT3–Th17-associated mechanism in Sjögren-related DED, and the local accumulation of S100A9-positive granulocytes and monocytes in anterior ocular inflammation. Competing metabolic, injury-related, and molecular inputs are included to emphasize that TLR4-associated signaling is not ligand-specific. Dark solid borders indicate context-specific direct support for the depicted claim component; dashed borders indicate model-associated evidence; dotted grey borders indicate indirect biological context; and pale grey transparency indicates limited or unresolved evidence. Created in BioRender. Wang, P. (2026) https://BioRender.com/ikblyuo (accessed on 30 June 2026).

Selected ocular models have reported S100A9- or S100A8/A9-associated responses, although the relevant molecular species and proposed mechanisms vary by disease context and cell type [10,11,12] (Figure 2). Shared limitations concerning source attribution, systemic transport, and ligand-specific receptor engagement are summarized in Table 1 and Section 7.

### 4.1. Context-Specific TLR4-Associated Evidence

Biochemical studies support TLR4 as a candidate receptor for selected extracellular S100A9-related molecular forms [28]. In an experimental DR model, endothelial cell-specific TLR4 deletion reduced vascular permeability, neuronal injury, and vascular degeneration, whereas Müller cell-specific deletion produced more limited effects [33]. This study provides direct model-specific evidence for cell-type-dependent retinal TLR4 function but does not demonstrate S100A9-specific receptor engagement.

In animal models of DR and lacrimal gland-excision DED, pharmacological pathway inhibition was associated with improvements in selected retinal or corneal outcomes and concurrent reductions in S100A9/TLR4-associated signals [10,11]. These findings are consistent with model-specific involvement of S100A9–TLR4-associated signaling but do not demonstrate direct ligand–receptor binding or in vivo receptor occupancy. In a Sjögren-related DED model, S100A8/A9 was implicated in aconitate decarboxylase 1 (Acod1)/signal transducer and activator of transcription 3 (STAT3)-associated dendritic cell activation and T helper 17 (Th17) responses [12], representing a distinct molecular species and proposed signaling mechanism.

### 4.2. Context Dependence and Local Ocular Amplification

Pathway activation alone does not establish ligand-specific target engagement because TLR4-associated inflammatory signaling can be influenced by multiple metabolic and tissue-injury stimuli. More direct evidence of local ocular cellular accumulation has been demonstrated in endotoxin-induced anterior ocular inflammation, in which S100A9-positive granulocytes and monocytes accumulated in the iris–ciliary body and cornea [13]. A separate study describing compartment-specific retinal and uveal myeloid responses to systemic inflammation [47] was not S100A9-specific and is included only as an indirect biological context. Collectively, these findings suggest that recruited ocular myeloid cells may contribute to local inflammatory amplification in selected models, although they do not establish the source of the relevant S100A9-related species or a ligand-specific receptor mechanism. Disease-specific evidence is examined in Section 5.

## 5. Compartment-Specific Ocular Evidence

### 5.1. Retina: Local S100A9-Associated Evidence and Hypothesized Systemic Contribution

**Figure 3:**
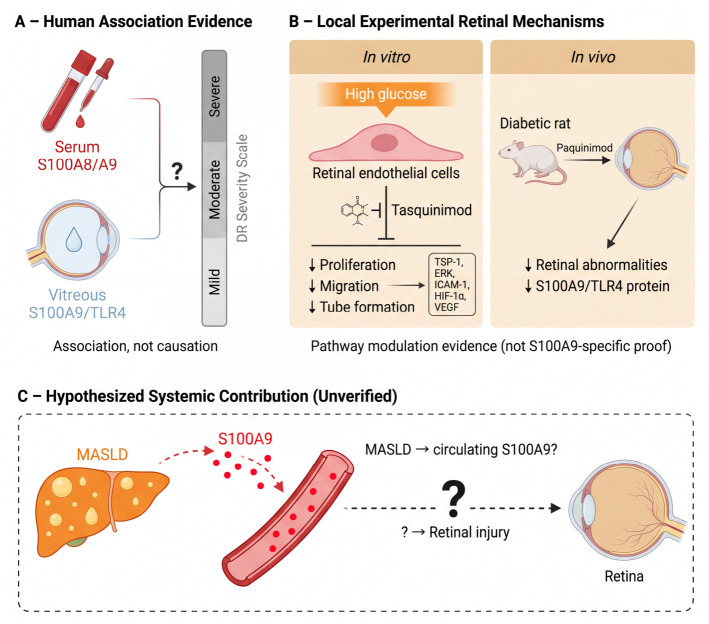
Human associations, experimental retinal evidence, and hypothesized systemic contribution of S100A9-related species in DR. Circulating S100A8 and S100A9 were separately associated with DR severity, whereas vitreous S100A9/TLR4-associated proteins represent an ocular compartment association without evidence of hepatic origin or S100A8/A9 heterocomplex activity. Experimental evidence comprises Paquinimod-associated improvement in diabetic rats and Tasquinimod-associated modulation of high glucose-treated human retinal endothelial cells; neither establishes S100A9-specific causality, direct target engagement, or hepatic mediation. Context-specific retinal and endothelial responses, therefore, have more direct support than a source-resolved systemic hepatic contribution, which remains hypothetical. Created in BioRender. Wang, P. (2026) https://BioRender.com/1tp9qhm (accessed on 30 June 2026).

Within the evidence considered in this review, findings linking S100A9-related species to DR comprise cross-sectional systemic and vitreous associations, one experimental DR study, and one retinal endothelial cell model [10,46,48] (Figure 3).

Associations between MASLD and DR remain inconsistent across observational studies and meta-analyses [4,5,6,7]. In patients with type 2 diabetes, circulating S100A8 and S100A9 concentrations were separately associated with DR severity [46], while increased S100A9/TLR4-associated proteins were reported in vitreous samples from patients with DR [10]. These findings document systemic and ocular compartment associations but do not establish S100A8/A9 heterocomplex activity, hepatic origin, or hepatic mediation.

In diabetic rats, Paquinimod treatment reduced selected retinal abnormalities, with concurrent reductions in S100A9/TLR4-associated protein expression [10]. In high glucose-treated human retinal endothelial cells, Tasquinimod reduced proliferation, migration, and tube formation and altered TSP-1-, ERK-, ICAM-1-, HIF-1α-, and VEGF-associated responses [48]. Because Tasquinimod also modulates HDAC4 signaling and suppressive myeloid cells in non-ocular cancer models [49,50], these studies provide model-specific animal and cellular evidence of pathway-associated modulation rather than S100A9-specific causal proof or direct target engagement.

Overall, current evidence provides more direct support for context-specific retinal and endothelial responses than for a source-resolved circulating contribution, which remains hypothetical and is summarized in Table 1.

### 5.2. Ocular Surface and DED: Local S100A9-Related Evidence

DED has been reported in selected liver-disease populations [8]. A primary biliary cholangitis cohort included coexisting seasonal allergic conjunctivitis, limiting attribution of the ocular surface findings to liver disease alone [51]. These clinical studies did not assess S100A9-related species and, therefore, support association rather than a shared molecular mechanism.

In a lacrimal gland-excision model, corneal epithelial S100A9 increased alongside TLR4 activation and impaired autophagic flux; pharmacological inhibition of S100A9 or TLR4 improved selected DED outcomes [11]. This provides model-specific animal evidence consistent with local S100A9–TLR4-associated activity but does not demonstrate direct ligand–receptor binding or in vivo receptor occupancy. In studies of Sjögren-related DED, increased S100A8/A9 in patient peripheral blood mononuclear cells and non-obese diabetic mouse lacrimal glands was implicated in Acod1/STAT3-associated dendritic cell activation and Th17 responses, while Paquinimod improved selected manifestations in mice [12]. This evidence concerns the S100A8/A9 complex rather than isolated S100A9.

**Figure 4:**
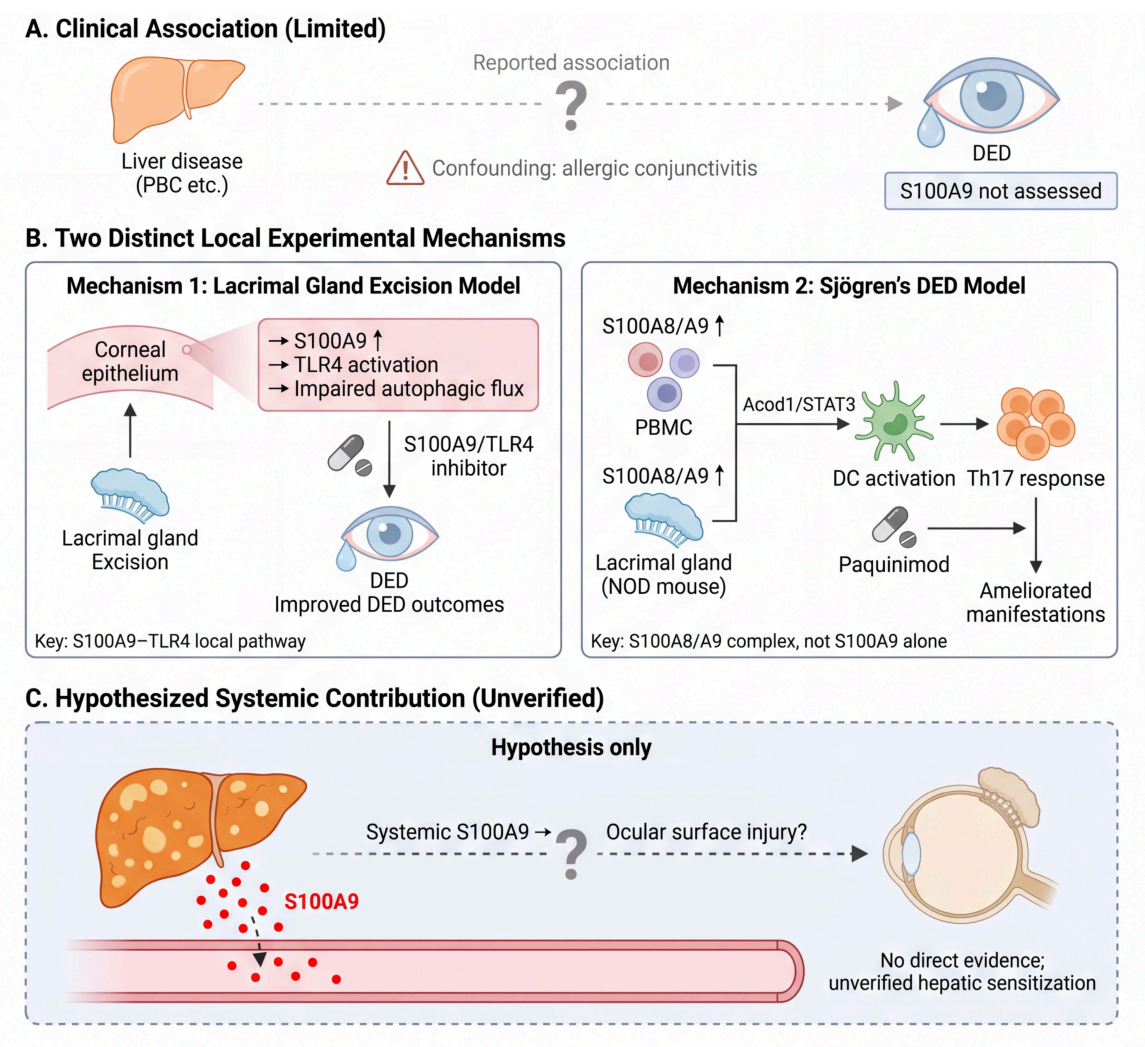
Clinical associations and model-specific local S100A9-related responses in DED. Clinical studies indicate an association between selected liver diseases and DED; however, these studies did not measure S100A9-related species and do not establish a shared molecular mechanism. Experimental studies provide model-specific support for two distinct candidate local response pathways: S100A9–TLR4-associated responses accompanied by impaired autophagic flux in a lacrimal gland-excision dry eye model, and S100A8/A9–Acod1/STAT3-associated dendritic cell and Th17 responses in Sjögren-related dry eye models. These findings involve different molecular species and experimental contexts and should not be interpreted as a unified pathway. A systemic S100A9-related contribution from liver disease to ocular surface injury remains a hypothesis only. Created in BioRender. Wang, P. (2026) https://BioRender.com/piu6dcr (accessed on 30 June 2026).

Current evidence, therefore, provides model-specific support for two distinct local response pathways rather than establishing a unified ocular surface mechanism. A systemic S100A9-related contribution remains a hypothesis only and is summarized in Table 1 (Figure 4).

### 5.3. Uveitis: Disease- and Model-Specific S100A9-Related Evidence

**Figure 5:**
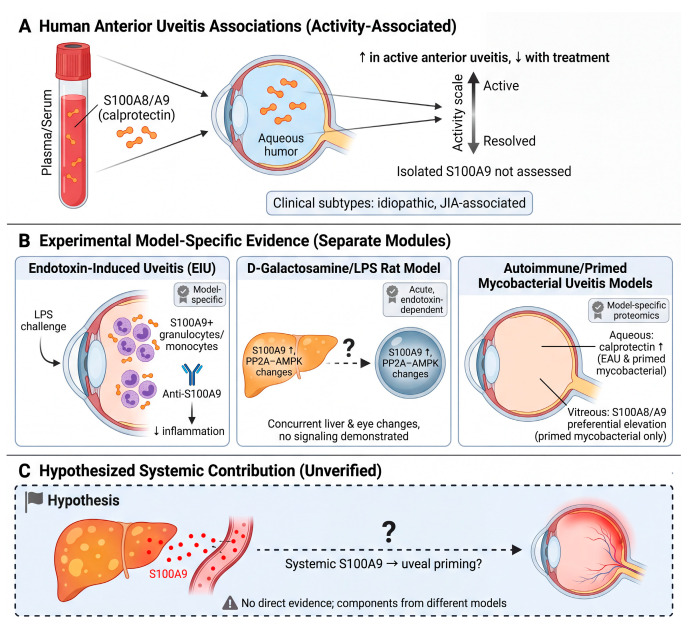
Human, ocular fluid, and experimental evidence for S100A9-related species in uveitis. Human studies show activity-associated increases in circulating or aqueous humor S100A8/A9-related analytes in selected anterior-uveitis populations, without establishing a subtype-independent biomarker, defined oligomeric form, or transport direction. Experimental evidence includes local accumulation of S100A9-positive granulocytes and monocytes and partial reductions in inflammation following anti-S100A9 treatment in EIU, model-specific ocular fluid changes in experimental autoimmune and primed mycobacterial uveitis, and concurrent hepatic and ocular alterations in an acute D-galactosamine/lipopolysaccharide rat model. These findings arise from distinct diseases and experimental systems and do not constitute a continuous hepatic-to-ocular pathway. A systemic sensitization contribution, therefore, remains hypothetical. Created in BioRender. Wang, P. (2026) https://BioRender.com/fha6tts (accessed on 30 June 2026).

Uveitis is heterogeneous in anatomical location and etiology; S100A9-related findings should therefore be interpreted within defined clinical subtypes and experimental models [52,53] (Figure 5).

Clinical studies primarily assessed S100A8/A9 or calprotectin immunoreactivity rather than isolated S100A9. Plasma S100A8/A9 was elevated in active acute anterior uveitis and declined with corticosteroid-associated clinical improvement [54]. In idiopathic acute anterior uveitis, serum calprotectin was increased and correlated with disease activity and macular thickness [55]. A pilot study found increased serum S100A8/A9 and S100A12 and increased aqueous humor S100A8/A9 in juvenile idiopathic arthritis-associated and idiopathic anterior uveitis [56]. These studies document activity-associated measurements in selected anterior-uveitis populations but do not establish a subtype-independent biomarker, a defined oligomeric species, or a source-resolved systemic contribution.

A D-galactosamine/lipopolysaccharide rat model showed concurrent hepatic and ocular S100A9-associated changes with protein phosphatase 2A (PP2A)–AMP-activated protein kinase (AMPK) alterations [57]. Because the model is acute and endotoxin-dependent, it provides same-model evidence of concurrent hepatic and ocular changes but does not establish hepatic-to-ocular signaling. In endotoxin-induced uveitis (EIU), S100A9-positive granulocytes and monocytes accumulated in the iris–ciliary body and cornea; anti-S100A9 antibody treatment partially reduced inflammation [13]. Comparative proteomics identified increased aqueous humor calprotectin in experimental autoimmune uveitis (EAU) and primed mycobacterial uveitis, with preferential vitreous S100A8 and S100A9 elevation in the latter model [58]. These findings provide disease- and model-specific evidence of ocular involvement by S100A9-related species; a source-resolved systemic contribution remains hypothetical and is summarized in Table 1.

### 5.4. Evidence-Integrated Working Model

Current evidence can be organized into three largely separate domains: disease-associated hepatic myeloid expression of S100A9 or S100A8/A9 [19,20,21,22,23,24,25,26,27]; circulating and ocular fluid associations involving S100A8, S100A9, or S100A8/A9 in selected retinal and anterior-uveitis populations [10,46,54,55,56]; and local abundance, cellular infiltration, or pharmacological pathway modulation in retinal, ocular surface, and uveitis models [10,11,12,13,48,58]. One acute D-galactosamine/lipopolysaccharide rat study additionally reported concurrent hepatic and ocular S100A9-associated changes [57] but did not resolve source, transport, or directional inter-organ signaling.

These findings are consistent with a consideration of S100A9-related species as context-dependent candidate nodes rather than components of a demonstrated continuous hepato–ocular pathway. Local ocular involvement has more direct support within specific models and compartments, whereas a source-resolved systemic contribution remains a hypothesis only. Table 1 summarizes the claim-support categories and principal inferential boundaries.

## 6. Preclinical Therapeutic Concepts

**Figure 6:**
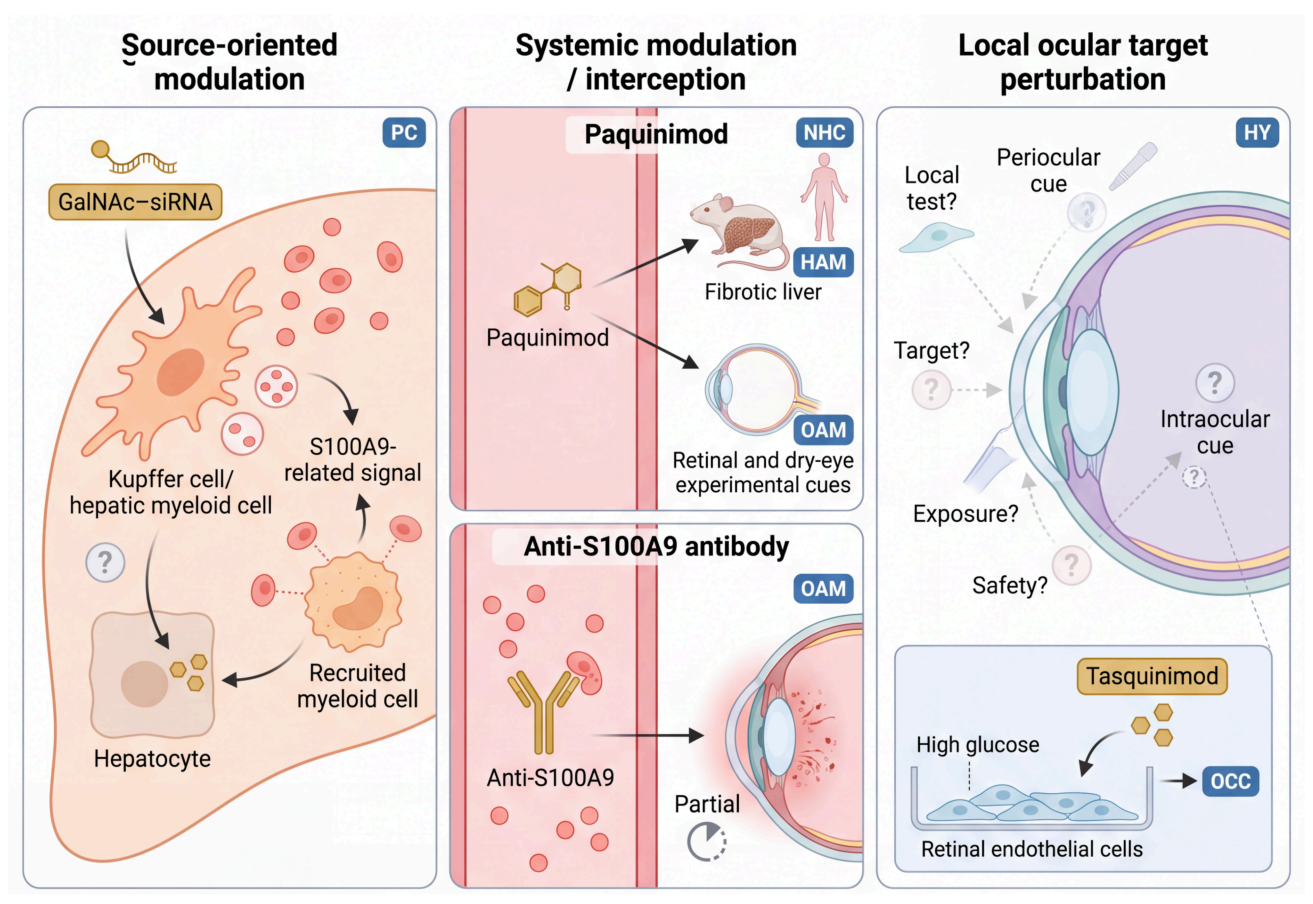
Experimental framework and evidence maturity of S100A9-related intervention concepts. Source-oriented modulation includes hepatocyte-directed GalNAc–siRNA as a delivery-platform concept rather than an S100A9-directed product; the prominent hepatic S100A9-related populations in the reviewed settings are myeloid rather than hepatocytic. Systemic evidence comprises separate Paquinimod studies in hepatic and ocular animal models, non-ophthalmic human exposure data, and partial anti-S100A9 effects in EIU; these findings do not establish source selectivity, S100A9-specific target engagement, or coordinated hepato–ocular efficacy. Tasquinimod evidence is limited to retinal endothelial cell culture and is complicated by pleiotropic pharmacology. No validated topical, periocular, or intraocular S100A9-directed formulation was identified in the studies reviewed in the literature; local ocular intervention, therefore, remains hypothetical. Black solid arrows indicate experimentally supported intervention concepts, whereas light-grey dashed arrows indicate hypothetical or unvalidated relationships. Created in BioRender. Wang, P. (2026) https://BioRender.com/00xwzib (accessed on 30 June 2026).

For hypothesis generation, the available evidence can be organized into three experimental intervention concepts: systemic modulation or interception, source-oriented modulation, and local ocular target perturbation. These concepts are intended to distinguish potential source, systemic, and local contributions rather than to define a clinical treatment sequence. Their developmental maturity is summarized in Table 2 and Figure 6.

### 6.1. Systemic Interception and Source-Oriented Concepts

A non-ocular mechanistic study identified S100A9 as a binding partner of selected quinoline-3-carboxamides [59]. Paquinimod reversed established fibrosis in a mouse liver model [60] and was associated with improvement in selected outcomes across experimental DR and DED models [10,11,12]. A repeat-dose study in systemic lupus erythematosus provides non-ophthalmic pharmacokinetic and tolerability data only [61]. Collectively, these findings provide preliminary evidence that S100A9-related extracellular pathways can be pharmacologically modulated in separate hepatic and ocular models; however, they do not demonstrate S100A9-specific target engagement or a coordinated hepato–ocular treatment effect.

Anti-S100A9 antibody treatment partially reduced inflammation in EIU [13], providing model-specific evidence from an experimental antibody intervention rather than validation of a therapeutic antibody platform or a clinically developable product. Hepatocyte-directed N-acetylgalactosamine (GalNAc)–small interfering RNA (siRNA) is an established hepatocyte-delivery platform [62]; however, the prominent hepatic S100A9-related populations identified in the reviewed settings are myeloid rather than hepatocytic [23,63]. A source-selective S100A9-silencing strategy, therefore, remains a platform-level concept; hepatocyte-directed delivery may not address the predominant candidate cellular sources.

### 6.2. Local Ocular Intervention Concepts

Within the studies reviewed in the literature, anti-S100A9 treatment in EIU provides the most direct ocular target-perturbation evidence, although the effect was partial and model-specific [13]. Systemic Paquinimod studies in retinal and dry eye models [10,11,12] and Tasquinimod-associated effects in high glucose-treated retinal endothelial cells [48] do not constitute local ocular delivery studies and do not establish ocular target engagement. Tasquinimod also has broader HDAC4-related and myeloid-modulatory actions demonstrated in non-ocular models [49,50,64].

No S100A9-directed topical, periocular, or intraocular formulation was identified in the studies reviewed in the literature. Any future local development program would need to define the targeted molecular species and evaluate ocular exposure, analyte-specific target modulation, dose response, reversibility, and local safety. Local S100A9-directed intervention should, therefore, be regarded as a hypothesis-testing development concept rather than an available or validated ocular treatment.

### 6.3. Safety, Target Specificity, and Translational Constraints

Non-ocular infectious and regenerative studies indicate context-dependent roles for S100A9-related species in host defense and tissue repair [15,18,65,66]. In one non-ocular tumor model, S100A9 blockade impaired the initiation of antitumor immunity [67], while advanced liver disease is independently associated with immune dysfunction and increased infection risk [68]. These observations indicate that future systemic inhibition strategies require evaluation of reversibility, infection susceptibility, leukocyte function, hepatic effects, and tissue-repair outcomes.

Molecular-form and modality specificity will be important because isolated S100A9, S100A8/A9 heterocomplexes, calcium-dependent heterotetramers, and extracellular vesicle-associated material should not be assumed to be biologically or pharmacologically interchangeable [15,28,29,31]. Observed pharmacological responses cannot be attributed specifically to S100A9 when the compounds also have pleiotropic molecular or cellular actions [49,50,64]; thus, antibody candidates would require characterization of Fragment crystallizable (Fc)-mediated functions [69]. Future development would, therefore, require analyte-specific evidence of target modulation at the minimum effective and compartment-appropriate exposure. Modality-specific requirements are summarized in Table 2 and Supplementary Table S3; causal-validation priorities are addressed in Section 7.

## 7. Discussion

Current evidence comprises disease-associated hepatic myeloid expression, circulating or ocular fluid associations, and local ocular responses. Together, these domains support hypothesis generation concerning S100A9-related species in hepato–ocular crosstalk; however, these arise largely from separate clinical and experimental systems.

### 7.1. Interpretation and Competing Explanations

The most direct available evidence provides context-specific support for local ocular involvement within selected diseases and experimental models, whereas a source-resolved systemic contribution remains inferential. Clinical liver–eye associations are heterogeneous across MASLD–DR, liver disease–DED, and anterior-uveitis populations [4,5,6,7,8,51,54,55,56]. Circulating measurements may reflect systemic myeloid activation, whereas ocular fluid or tissue signals may arise locally [10,11,12,13,46]. Cross-sectional studies cannot establish whether these analytes precede, accompany, or follow ocular disease.

Interpretation also depends on the measured molecular species and biological matrix. S100A9, S100A8/A9 complexes, higher-order assemblies, phosphorylated forms, and extracellular vesicle-associated material should not be treated as analytically or biologically interchangeable; serum, plasma, ocular fluid, and tissue measurements require matrix-specific interpretation [15,18,28,29,30,31,32]. In addition, the available pharmacological studies involve model-specific and partly pleiotropic interventions [10,11,12,13,48,49,50,59,60]. These studies provide preliminary evidence that S100A9-related or overlapping inflammatory pathways can be pharmacologically modulated in selected models, but they do not establish S100A9-specific target engagement, causal dominance, or clinical tractability. S100A9-related species may, therefore, be more appropriately considered as context-dependent candidate nodes rather than as components of a universal linear pathway.

### 7.2. Causal and Clinical Validation Priorities

Future experimental validation should define the relevant molecular species, resolve their cellular and organ sources, and measure systemic exposure, ocular distribution or target modulation, and prespecified ocular outcomes within the same model. Where technically feasible, source-selective perturbation should be compared with systemic myeloid and local ocular interventions, with rescue or reversal experiments included when appropriate. The D-galactosamine/lipopolysaccharide model provides a same-model example of concurrent hepatic and ocular changes but remains hypothesis-generating because a systemic endotoxin may activate both organs in parallel [57].

Clinical studies should use prospective, diagnosis-specific cohorts with standardized ocular phenotyping, serial analyte-specific measurements, and prespecified adjustment for metabolic and inflammatory confounders. Paired blood and ocular fluid sampling may help to distinguish systemic from local signals, although invasive sampling should be limited to clinically justified procedures [70]. Tear studies require standardized collection, storage, processing, and analysis [71,72]. Biomarker-development studies should prespecify the intended context of use and separately evaluate analytical validity, clinical validity, and incremental clinical utility [32,73,74].

Ethical oversight should follow applicable research ethics principles [70]. Data minimization, pseudonymization, access control, retention, and data transfer should follow the relevant data-protection framework, such as Regulation (EU) 2016/679, where applicable [75]. Interventional studies should follow current good clinical-practice requirements, including risk-proportionate data governance and monitoring [76].

### 7.3. Limitations of the Review and Evidence Base

This SANRA-informed article is a structured narrative and hypothesis-generating review rather than a systematic review [38]. The searches were iterative and claim-directed; therefore, no exhaustive study set, formal screening count, study-level risk-of-bias assessment, meta-analysis, or GRADE-based certainty evaluation was undertaken. The claim-support categories describe evidence directness and coherence rather than study quality or formal certainty. This approach may have introduced selection bias toward mechanistically coherent or frequently cited evidence, despite efforts to consider conflicting and null findings, when identified.

The evidence base remains heterogeneous in disease context, molecular species, biological matrix, assay, model, and intervention. Human studies are predominantly observational or exploratory, whereas hepatic expression, systemic measurements, ocular findings, and intervention effects have generally been examined in separate study systems. The scope of this review is limited to DR, DED, and selected forms of uveitis. The principal inferential boundaries are summarized in Table 1, with study-level details provided in Supplementary Table S2.

## 8. Conclusions

Current evidence provides context-specific support for disease-associated hepatic myeloid expression of S100A9-related species and documents selected ocular associations and model-specific responses. However, no study has demonstrated a continuous causal sequence from hepatic production through systemic transport and ocular target engagement to modification of an ocular phenotype. S100A9-related species may, therefore, be more appropriately regarded as context-dependent candidate nodes rather than a confirmed molecular bridge. Their biomarker and therapeutic relevance remains exploratory and requires source-resolved, analyte-specific validation in integrated experimental studies and prospective clinical cohorts.

## Figures and Tables

**Table 1:** Claim-level appraisal of the proposed S100A9-related hepato–ocular framework.

Claim Domain	Evidence Category	Concise Interpretation
Hepatic expression	CD	Human and experimental studies provide context-specific support for disease-associated S100A9-, S100A8-, or S100A8/A9-related expression predominantly in hepatic myeloid populations. These findings do not establish extracellular release or quantify the liver’s contribution to circulating analytes.
Release and potential systemic availability	LI	Mechanistic and cross-tissue studies provide context-specific evidence for several released or circulating forms, including phosphorylated S100A9, S100A8/A9 associated with gasdermin D pores or NETs, and extracellular vesicle-associated material. Their quantitative relevance to liver disease, hepatic origin, and ocular delivery remains unresolved.
Hepatic-to-ocular transport or systemic contribution	HO	No study has connected hepatic production, source-resolved systemic release, ocular biodistribution, receptor engagement, and modification of an ocular phenotype within one experimental sequence.
Ocular receptor competence and TLR4-associated responsiveness	CD/LI	Selected retinal and corneal models provide cell- and model-specific evidence of TLR4 competence and S100A9–TLR4-associated responses. Direct S100A9-specific receptor binding or in vivo occupancy has not been demonstrated, and ocular S100A9–RAGE evidence remains indirect.
Retinal evidence	CD/LI	Human blood and vitreous findings are associative and source-unresolved, whereas animal and cell studies provide model-specific evidence of retinal or endothelial responsiveness. Hepatic mediation, temporal prediction, and S100A9-specific target engagement have not been established.
Ocular surface and dry eye evidence	CD/LI	Clinical liver disease–DED associations did not measure S100A9-related mediators. Separate models provide evidence consistent with local S100A9–TLR4-associated and S100A8/A9–Acod1/STAT3-associated response pathways, which should not be merged into a single mechanism.
Uveitis evidence	CD/LI	Human plasma, serum, and aqueous-humor findings are activity-associated and subtype-specific. Animal models provide context-specific evidence of local myeloid involvement and model-dependent ocular abundance but do not establish an integrated liver-to-uvea pathway.
Biomarker and translational implications	LI/HO	Assay studies demonstrate that S100A9-related analytes can be measured, whereas preclinical interventions provide limited evidence of experimental tractability in selected models. No analyte has demonstrated incremental clinical utility, and no source-selective or locally delivered S100A9-directed intervention has established hepato–ocular efficacy.

Mixed labels indicate that different components of a claim are supported at different levels. These categories describe claim-level directness and coherence but not study quality, formal risk of bias, quantitative certainty, or GRADE ratings. Detailed study types, reference numbers, disease and experimental contexts, molecular species, and principal limitations are provided in Supplementary Table S2.

**Table 2:** Developmental maturity of S100A9-related intervention concepts.

Intervention Concept	Evidence Stage	Current Interpretation and Principal Gap
Paquinimod-mediated systemic modulation	OAM + HAM + NHC	Supports systemic pharmacological tractability across separate ocular, hepatic, and non-ophthalmic human settings. Source selectivity, ophthalmic dosing, ocular target engagement, and coordinated hepato–ocular efficacy remain unestablished.
Tasquinimod-mediated pathway modulation	OCC	Supports retinal endothelial activity in cell culture. S100A9-specific target engagement, ocular pharmacokinetics, local safety, and ophthalmic in vivo efficacy have not been demonstrated.
Experimental anti-S100A9 antibody intervention	OAM	Provides acute animal-model target-perturbation evidence in EIU. Molecular-form and epitope specificity, ocular exposure, Fc-mediated effects, and chronic-disease relevance remain unresolved.
Hepatic or myeloid source-directed modulation	PC	Hepatocyte-directed RNA delivery is a platform concept; relevant hepatic myeloid populations have been mapped. No source-selective S100A9 product exists; hepatocyte targeting may not address predominant myeloid sources, whereas broad myeloid targeting lacks organ specificity.
Local ocular S100A9-directed intervention	HY	No validated topical, periocular, or intraocular S100A9-directed formulation was identified. The target molecular species, ocular pharmacokinetics or biodistribution, target modulation, dose response, reversibility, and local host-defense and repair effects remain undefined.

Evidence-stage labels describe the source and developmental maturity of the available evidence rather than treatment efficacy, study quality, formal risk of bias, quantitative certainty, or GRADE ratings. Representative studies, reference numbers, routes, and modality-specific limitations are provided in Supplementary Table S3.

## Data Availability

No new data were created or analyzed in this study. Data sharing is not applicable to this article.

## Supplementary Materials

The following supporting information can be downloaded at: https://www.mdpi.com/article/10.3390/biology15151306/s1, Supplementary Text S1: Additional Information on Review Methodology; Supplementary Table S1: Literature Search Strategies and Evidence-Identification Framework; Supplementary Table S2: Study-Level Evidence Matrix Underpinning the Claim-Level Appraisal in Table 1; Supplementary Table S3: Representative Evidence and Developmental Boundaries of Experimental S100A9-Related Intervention Concepts. All references cited in Supplementary Text S1 and Supplementary Tables S1–S3 are also cited in the main text and included in the main reference list [8,10,11,12,13,15,18,19,20,21,22,23,24,25,26,27,28,29,30,31,32,33,34,35,36,38,40,41,42,43,44,46,48,49,50,51,54,55,56,57,58,59,60,61,62,63,64,65,66,67,68,69].

## Abbreviations

The following abbreviations are used in this manuscript:

Acod1	Aconitate decarboxylase 1
AMPK	AMP-activated protein kinase
CD	Context-specific direct support
DED	Dry eye disease
DR	Diabetic retinopathy
EAU	Experimental autoimmune uveitis
EIU	Endotoxin-induced uveitis
EV	Extracellular vesicle
Fc	Fragment crystallizable
GalNAc	N-acetylgalactosamine
GRADE	Grading of Recommendations Assessment, Development, and Evaluation
HAM	Hepatic animal-model evidence
HO	Hypothesis only (claim-support category)
HY	Hypothesis only (intervention-development category)
LI	Limited or indirect support
MASLD	Metabolic dysfunction-associated steatotic liver disease
MASH	Metabolic dysfunction-associated steatohepatitis
MetALD	Metabolic dysfunction and alcohol-associated liver disease
NET	Neutrophil extracellular trap
NHC	Non-ophthalmic human clinical exposure
OAM	Ocular animal-model evidence
OCC	Ocular cell-culture evidence
PC	Platform concept
PP2A	Protein phosphatase 2A
RAGE	Receptor for advanced glycation end products
S100A9	S100 calcium-binding protein A9
SANRA	Scale for the Assessment of Narrative Review Articles
siRNA	Small interfering RNA
STAT3	Signal transducer and activator of transcription 3
Th17	T helper 17
TLR4	Toll-like receptor 4
