# Supplementary material for: S100A9 as a Candidate Molecular Bridge in Hepato–Ocular Crosstalk
Source: Biology (Basel). 2026 Aug 5;15(15):1306. doi: 10.3390/biology15151306 (PMC13464601; doi:10.3390/biology15151306)
Supplement: Supplementary file 1 [file biology-15-01306-s001.zip › Supplementary Table S1 Literature Search Strategies and Evidence-Identification Framework.pdf]

Supplementary Table S1. Literature Search Strategies and Evidence-Identification Framework

The searches were iterative, targeted, and claim-directed rather than exhaustive. The supplementary methodological text and tables below report the evidence-selection and claim-level appraisal procedures, search administration, exact concept blocks, database-specific field syntax, Boolean combinations, limits, legacy terminology, and duplicate-handling procedures used for the review. Because no fixed screening dataset was established, no PRISMA-style flow count or category-specific study count was maintained.

Table S1A. Information sources, coverage, fields, limits, and final search date

Information source	Platform	Coverage and fields	Limits applied at search stage	Final search date	Purpose
PubMed/MEDLINE	PubMed	Database inception; free-text terms searched in Title/Abstract fields	No date, language, species, or document-type filter	1 May 2026	Biomedical, clinical, animal, cellular, molecular, structural, biomarker, and intervention studies
Embase	Elsevier Embase	Database inception; free-text terms searched in title and abstract fields (ti,ab)	No date, language, species, or document-type filter	1 May 2026	Expanded biomedical, pharmacological, European, and conference-indexed retrieval
Web of Science Core Collection	Clarivate	Database inception; Topic field (TS: title, abstract, author keywords, and Keywords Plus)	No date, language, species, or document-type filter	1 May 2026	Interdisciplinary retrieval and backward/forward citation tracking
Scopus	Elsevier	Database inception; TITLE-ABS-KEY fields	No date, language, species, or document-type filter	1 May 2026	Broad interdisciplinary retrieval and forward-citation tracking
Reference-list examination	Reference lists of key primary studies and focused reviews	Not applicable	Primary studies were prioritized; reviews were used mainly for contextual framing and identification of primary literature	1 May 2026	Identification of directly relevant studies not retrieved in the targeted database modules
Citation tracking	Web of Science, Scopus, and database-linked citation tools	Not applicable	Applied to key hepatic, ocular, biomarker, molecular-form, and intervention reports	1 May 2026	Identification of subsequent or linked studies relevant to specific claims

Legacy terminology used for retrieval: NAFLD, NASH, nonalcoholic fatty liver disease, non-alcoholic fatty liver disease, nonalcoholic steatohepatitis, non-alcoholic steatohepatitis, MAFLD, and metabolic-associated fatty liver disease were retained alongside MASLD, MASH, and MetALD. Legacy terms were used for retrieval only and do not supersede the terminology policy used in the manuscript.

Table S1B. Exact search-concept blocks

Block	Concept	Exact free-text terms
**S**	S100A9-related molecular species	"S100A9" OR "S100A8/A9" OR "S100A8-S100A9" OR calprotectin OR "MRP8/MRP14" OR "MRP8/14" OR "myeloid-related protein 8/14"
**H**	Hepatic context	liver OR hepatic OR MASLD OR "metabolic dysfunction-associated steatotic liver disease" OR MASH OR "metabolic dysfunction-associated steatohepatitis" OR NAFLD OR "nonalcoholic fatty liver disease" OR "non-alcoholic fatty liver disease" OR NASH OR "nonalcoholic steatohepatitis" OR "non-alcoholic steatohepatitis" OR MAFLD OR "metabolic-associated fatty liver disease" OR MetALD OR "metabolic dysfunction and alcohol-associated liver disease" OR "alcohol-related liver disease" OR cirrhosis OR "hepatic fibrosis" OR "drug-induced liver injury" OR DILI OR "ischemia-reperfusion injury" OR "ischaemia-reperfusion injury" OR "viral hepatitis"
**O**	Ocular context	eye OR ocular OR retina OR retinal OR "diabetic retinopathy" OR "retinal pigment epithelium" OR RPE OR "retinal endothelial" OR "blood-retinal barrier" OR cornea OR corneal OR "ocular surface" OR "dry eye" OR "dry eye disease" OR "lacrimal gland" OR uvea OR uveal OR uveitis OR "acute anterior uveitis" OR "experimental autoimmune uveitis" OR "endotoxin-induced uveitis"
**M**	Receptor and signaling context	TLR4 OR "Toll-like receptor 4" OR RAGE OR "receptor for advanced glycation end products" OR "NF-kappa B" OR "NF-κB" OR MAPK OR HIF-1alpha OR HIF-1α OR VEGF OR NLRP3 OR inflammasome OR PP2A OR AMPK OR autophagy OR STAT3 OR Acod1
**F**	Molecular form and release	heterodimer OR heterotetramer OR tetramer OR oligomerization OR "calcium binding" OR phosphorylation OR Thr113 OR secretion OR release OR "gasdermin D" OR GSDMD OR "neutrophil extracellular trap" OR NET OR "extracellular vesicle" OR exosome
**B**	Biomarker and matrix context	biomarker OR serum OR plasma OR circulating OR tear OR "aqueous humor" OR "aqueous humour" OR vitreous OR "ocular fluid" OR ELISA OR immunoturbidimetry OR "multiplex immunoassay" OR prognostic OR predictive OR monitoring
**T**	Intervention and translational context	Paquinimod OR Tasquinimod OR "anti-S100A9" OR "S100A9 antibody" OR "neutralizing antibody" OR siRNA OR "small interfering RNA" OR GalNAc OR "gene silencing" OR "target engagement" OR pharmacokinetics OR therapeutic OR treatment
**C**	hATTR comparator	"hereditary transthyretin amyloidosis" OR hATTR OR "transthyretin amyloidosis" OR "ocular amyloidosis" OR "vitreous amyloidosis"

Block	Concept	Exact free-text terms
**L**	hATTR source-attribution terms	"liver transplantation" OR ocular OR vitreous OR retina OR glaucoma

Table S1C. Search modules run separately during the iterative retrieval

Query	Search module	Exact Boolean combination	Primary purpose
Q1	Hepatic expression and cellular sources	(S) AND (H)	Hepatic expression, cell sources, local injury, fibrosis, toxic/infectious injury, and ischemia-reperfusion
Q2	Ocular expression and responsiveness	(S) AND (O)	Retinal, ocular-surface, lacrimal, corneal, and uveitis evidence
Q3	Direct or integrated hepato-ocular evidence	(S) AND (H) AND (O)	Studies evaluating hepatic and ocular findings in the same clinical or experimental setting
Q4	Molecular forms and release	(S) AND (F) AND ((H) OR (O))	Oligomeric forms, phosphorylation, secretion, gasdermin D, NETs, and extracellular vesicles
Q5	Ocular receptor and signaling evidence	(S) AND (O) AND (M)	Ocular S100A9- or S100A8/A9-associated receptor and downstream signaling studies
Q6	Circulating and ocular-fluid biomarkers	(S) AND ((H) OR (O)) AND (B)	Serum, plasma, tear, aqueous-humor, vitreous, and tissue biomarker studies
Q7	Intervention and target perturbation	(S) AND ((H) OR (O)) AND (T)	Pharmacological, antibody, gene-silencing, target-engagement, and pharmacokinetic studies
Q8	Clinical liver-eye associations	(H) AND (O)	Disease-specific liver-eye epidemiology, including studies not measuring S100A9
Q9	hATTR comparator	(C) AND (L)	Hepatic versus local ocular transthyretin source attribution

Table S1D. Database-specific implementation of the exact concept blocks

Each query module in Table S1C was run separately. The exact free-text terms were those listed in Table S1B, translated into the field syntax below. No database-level date, language, species, or document-type filter was applied. Searches were refreshed through 1 May 2026.

PubMed/MEDLINE

Fields: [Title/Abstract]. Limits: none. Final search date: 1 May 2026.

Block and modules	Exact database-specific block
**S (Q1-Q7)**	"S100A9"[Title/Abstract] OR "S100A8/A9"[Title/Abstract] OR "S100A8-S100A9"[Title/Abstract] OR calprotectin[Title/Abstract] OR "MRP8/MRP14"[Title/Abstract] OR "MRP8/14"[Title/Abstract] OR "myeloid-related protein 8/14"[Title/Abstract]
**H (Q1,Q3,Q4,Q6-Q8)**	liver[Title/Abstract] OR hepatic[Title/Abstract] OR MASLD[Title/Abstract] OR "metabolic dysfunction-associated steatotic liver disease"[Title/Abstract] OR MASH[Title/Abstract] OR "metabolic dysfunction-associated steatohepatitis"[Title/Abstract] OR NAFLD[Title/Abstract] OR "nonalcoholic fatty liver disease"[Title/Abstract] OR "non-alcoholic fatty liver disease"[Title/Abstract] OR NASH[Title/Abstract] OR "nonalcoholic steatohepatitis"[Title/Abstract] OR "non-alcoholic steatohepatitis"[Title/Abstract] OR MAFLD[Title/Abstract] OR "metabolic-associated fatty liver disease"[Title/Abstract] OR MetALD[Title/Abstract] OR "metabolic dysfunction and alcohol-associated liver disease"[Title/Abstract] OR "alcohol-related liver disease"[Title/Abstract] OR cirrhosis[Title/Abstract] OR "hepatic fibrosis"[Title/Abstract] OR "drug-induced liver injury"[Title/Abstract] OR DILI[Title/Abstract] OR "ischemia-reperfusion injury"[Title/Abstract] OR "ischaemia-reperfusion injury"[Title/Abstract] OR "viral hepatitis"[Title/Abstract]
**O (Q2-Q8)**	eye[Title/Abstract] OR ocular[Title/Abstract] OR retina[Title/Abstract] OR retinal[Title/Abstract] OR "diabetic retinopathy"[Title/Abstract] OR "retinal pigment epithelium"[Title/Abstract] OR RPE[Title/Abstract] OR "retinal endothelial"[Title/Abstract] OR "blood-retinal barrier"[Title/Abstract] OR cornea[Title/Abstract] OR corneal[Title/Abstract] OR "ocular surface"[Title/Abstract] OR "dry eye"[Title/Abstract] OR "dry eye disease"[Title/Abstract] OR "lacrimal gland"[Title/Abstract] OR uvea[Title/Abstract] OR uveal[Title/Abstract] OR uveitis[Title/Abstract] OR "acute anterior uveitis"[Title/Abstract] OR "experimental autoimmune uveitis"[Title/Abstract] OR "endotoxin-induced uveitis"[Title/Abstract]
**M (Q5)**	TLR4[Title/Abstract] OR "Toll-like receptor 4"[Title/Abstract] OR RAGE[Title/Abstract] OR "receptor for advanced glycation end products"[Title/Abstract] OR "NF-kappa B"[Title/Abstract] OR "NF-κB"[Title/Abstract] OR MAPK[Title/Abstract] OR HIF-1alpha[Title/Abstract] OR HIF-1α[Title/Abstract] OR VEGF[Title/Abstract] OR NLRP3[Title/Abstract] OR inflammasome[Title/Abstract] OR PP2A[Title/Abstract] OR AMPK[Title/Abstract] OR autophagy[Title/Abstract] OR STAT3[Title/Abstract] OR Acod1[Title/Abstract]
**F (Q4)**	heterodimer[Title/Abstract] OR heterotetramer[Title/Abstract] OR tetramer[Title/Abstract] OR oligomerization[Title/Abstract] OR "calcium binding"[Title/Abstract] OR phosphorylation[Title/Abstract] OR Thr113[Title/Abstract] OR secretion[Title/Abstract] OR release[Title/Abstract] OR "gasdermin D"[Title/Abstract] OR GSDMD[Title/Abstract] OR "neutrophil extracellular trap"[Title/Abstract] OR NET[Title/Abstract] OR "extracellular vesicle"[Title/Abstract] OR exosome[Title/Abstract]
**B (Q6)**	biomarker[Title/Abstract] OR serum[Title/Abstract] OR plasma[Title/Abstract] OR circulating[Title/Abstract] OR tear[Title/Abstract] OR "aqueous humor"[Title/Abstract] OR "aqueous humour"[Title/Abstract] OR vitreous[Title/Abstract] OR "ocular fluid"[Title/Abstract] OR ELISA[Title/Abstract] OR immunoturbidimetry[Title/Abstract] OR "multiplex immunoassay"[Title/Abstract] OR prognostic[Title/Abstract] OR predictive[Title/Abstract] OR monitoring[Title/Abstract]
**T (Q7)**	Paquinimod[Title/Abstract] OR Tasquinimod[Title/Abstract] OR "anti-S100A9"[Title/Abstract] OR "S100A9 antibody"[Title/Abstract] OR "neutralizing antibody"[Title/Abstract] OR siRNA[Title/Abstract] OR "small interfering RNA"[Title/Abstract] OR GalNAc[Title/Abstract] OR "gene silencing"[Title/Abstract] OR "target engagement"[Title/Abstract] OR pharmacokinetics[Title/Abstract] OR therapeutic[Title/Abstract] OR treatment[Title/Abstract]
**C (Q9)**	"hereditary transthyretin amyloidosis"[Title/Abstract] OR hATTR[Title/Abstract] OR "transthyretin amyloidosis"[Title/Abstract] OR "ocular amyloidosis"[Title/Abstract] OR "vitreous amyloidosis"[Title/Abstract]

Block and modules	Exact database-specific block
L (Q9)	"liver transplantation"[Title/Abstract] OR ocular[Title/Abstract] OR vitreous[Title/Abstract] OR retina[Title/Abstract] OR glaucoma[Title/Abstract]

Exact query history: Q1=(S) AND (H); Q2=(S) AND (O); Q3=(S) AND (H) AND (O); Q4=(S) AND (F) AND ((H) OR (O)); Q5=(S) AND (O) AND (M); Q6=(S) AND ((H) OR (O)) AND (B); Q7=(S) AND ((H) OR (O)) AND (T); Q8=(H) AND (O); Q9=(C) AND (L)

Embase

Fields: :ti,ab. Limits: none. Final search date: 1 May 2026.

Block and modules	Exact database-specific block
**S (Q1-Q7)**	'S100A9':ti,ab OR 'S100A8/A9':ti,ab OR 'S100A8-S100A9':ti,ab OR calprotectin:ti,ab OR 'MRP8/MRP14':ti,ab OR 'MRP8/14':ti,ab OR 'myeloid-related protein 8/14':ti,ab
**H (Q1,Q3,Q4,Q6-Q8)**	liver:ti,ab OR hepatic:ti,ab OR masld:ti,ab OR 'metabolic dysfunction-associated steatotic liver disease':ti,ab OR mash:ti,ab OR 'metabolic dysfunction-associated steatohepatitis':ti,ab OR nafld:ti,ab OR 'nonalcoholic fatty liver disease':ti,ab OR 'non-alcoholic fatty liver disease':ti,ab OR nash:ti,ab OR 'nonalcoholic steatohepatitis':ti,ab OR 'non-alcoholic steatohepatitis':ti,ab OR mafld:ti,ab OR 'metabolic-associated fatty liver disease':ti,ab OR metald:ti,ab OR 'metabolic dysfunction and alcohol-associated liver disease':ti,ab OR 'alcohol-related liver disease':ti,ab OR cirrhosis:ti,ab OR 'hepatic fibrosis':ti,ab OR 'drug-induced liver injury':ti,ab OR dili:ti,ab OR 'ischemia-reperfusion injury':ti,ab OR 'ischaemia-reperfusion injury':ti,ab OR 'viral hepatitis':ti,ab
**O (Q2-Q8)**	eye:ti,ab OR ocular:ti,ab OR retina:ti,ab OR retinal:ti,ab OR 'diabetic retinopathy':ti,ab OR 'retinal pigment epithelium':ti,ab OR rpe:ti,ab OR 'retinal endothelial':ti,ab OR 'blood-retinal barrier':ti,ab OR cornea:ti,ab OR corneal:ti,ab OR 'ocular surface':ti,ab OR 'dry eye':ti,ab OR 'dry eye disease':ti,ab OR 'lacrimal gland':ti,ab OR uvea:ti,ab OR uveal:ti,ab OR uveitis:ti,ab OR 'acute anterior uveitis':ti,ab OR 'experimental autoimmune uveitis':ti,ab OR 'endotoxin-induced uveitis':ti,ab
**M (Q5)**	tlr4:ti,ab OR 'Toll-like receptor 4':ti,ab OR rage:ti,ab OR 'receptor for advanced glycation end products':ti,ab OR 'NF-kappa B':ti,ab OR 'NF-κB':ti,ab OR mapk:ti,ab OR hif-1alpha:ti,ab OR hif-1α:ti,ab OR vegf:ti,ab OR nlrp3:ti,ab OR inflammasome:ti,ab OR pp2a:ti,ab OR ampk:ti,ab OR autophagy:ti,ab OR stat3:ti,ab OR acod1:ti,ab
**F (Q4)**	heterodimer:ti,ab OR heterotetramer:ti,ab OR tetramer:ti,ab OR oligomerization:ti,ab OR 'calcium binding':ti,ab OR phosphorylation:ti,ab OR thr113:ti,ab OR secretion:ti,ab OR release:ti,ab OR 'gasdermin D':ti,ab OR gsdmd:ti,ab OR 'neutrophil extracellular trap':ti,ab OR net:ti,ab OR 'extracellular vesicle':ti,ab OR exosome:ti,ab
**B (Q6)**	biomarker:ti,ab OR serum:ti,ab OR plasma:ti,ab OR circulating:ti,ab OR tear:ti,ab OR 'aqueous humor':ti,ab OR 'aqueous humour':ti,ab OR vitreous:ti,ab OR 'ocular fluid':ti,ab OR elisa:ti,ab OR immunoturbidimetry:ti,ab OR 'multiplex immunoassay':ti,ab OR prognostic:ti,ab OR predictive:ti,ab OR monitoring:ti,ab
**T (Q7)**	paquinimod:ti,ab OR tasquinimod:ti,ab OR 'anti-S100A9':ti,ab OR 'S100A9 antibody':ti,ab OR 'neutralizing antibody':ti,ab OR sirna:ti,ab OR 'small interfering RNA':ti,ab OR galnac:ti,ab OR 'gene silencing':ti,ab OR 'target engagement':ti,ab OR pharmacokinetics:ti,ab OR therapeutic:ti,ab OR treatment:ti,ab
**C (Q9)**	'hereditary transthyretin amyloidosis':ti,ab OR hattr:ti,ab OR 'transthyretin amyloidosis':ti,ab OR 'ocular amyloidosis':ti,ab OR 'vitreous amyloidosis':ti,ab
**L (Q9)**	'liver transplantation':ti,ab OR ocular:ti,ab OR vitreous:ti,ab OR retina:ti,ab OR glaucoma:ti,ab

Exact query history: Q1=(S) AND (H); Q2=(S) AND (O); Q3=(S) AND (H) AND (O); Q4=(S) AND (F) AND ((H) OR (O)); Q5=(S) AND (O) AND (M); Q6=(S) AND ((H) OR (O)) AND (B); Q7=(S) AND ((H) OR (O)) AND (T); Q8=(H) AND (O); Q9=(C) AND (L)

Web of Science Core Collection

Fields: TS=(...). Limits: none. Final search date: 1 May 2026.

Block and modules	Exact database-specific block
**S (Q1-Q7)**	TS=("S100A9" OR "S100A8/A9" OR "S100A8-S100A9" OR calprotectin OR "MRP8/MRP14" OR "MRP8/14" OR "myeloid-related protein 8/14")
**H (Q1,Q3,Q4,Q6-Q8)**	TS=(liver OR hepatic OR MASLD OR "metabolic dysfunction-associated steatotic liver disease" OR MASH OR "metabolic dysfunction-associated steatohepatitis" OR NAFLD OR "nonalcoholic fatty liver disease" OR "non-alcoholic fatty liver disease" OR NASH OR "nonalcoholic steatohepatitis" OR "non-alcoholic steatohepatitis" OR MAFLD OR "metabolic-associated fatty liver disease" OR MetALD OR "metabolic dysfunction and alcohol-associated liver disease" OR "alcohol-related liver disease" OR cirrhosis OR "hepatic fibrosis" OR "drug-induced liver injury" OR DILI OR "ischemia-reperfusion injury" OR "ischaemia-reperfusion injury" OR "viral hepatitis")
**O (Q2-Q8)**	TS=(eye OR ocular OR retina OR retinal OR "diabetic retinopathy" OR "retinal pigment epithelium" OR RPE OR "retinal endothelial" OR "blood-retinal barrier" OR cornea OR corneal OR "ocular surface" OR "dry eye" OR "dry eye disease" OR "lacrimal gland" OR uvea OR uveal OR uveitis OR "acute anterior uveitis" OR "experimental autoimmune uveitis" OR "endotoxin-induced uveitis")
**M (Q5)**	TS=(TLR4 OR "Toll-like receptor 4" OR RAGE OR "receptor for advanced glycation end products" OR "NF-kappa B" OR "NF-κB" OR MAPK OR HIF-1alpha OR HIF-1α OR VEGF OR NLRP3 OR inflammasome OR PP2A OR AMPK OR autophagy OR STAT3 OR Acod1)
**F (Q4)**	TS=(heterodimer OR heterotetramer OR tetramer OR oligomerization OR "calcium binding" OR phosphorylation OR Thr113 OR secretion OR release OR "gasdermin D" OR GSDMD OR "neutrophil extracellular trap" OR NET OR "extracellular vesicle" OR exosome)
**B (Q6)**	TS=(biomarker OR serum OR plasma OR circulating OR tear OR "aqueous humor" OR "aqueous humour" OR vitreous OR "ocular fluid" OR ELISA OR immunoturbidimetry OR "multiplex immunoassay" OR prognostic OR predictive OR monitoring)
**T (Q7)**	TS=(Paquinimod OR Tasquinimod OR "anti-S100A9" OR "S100A9 antibody" OR "neutralizing antibody" OR siRNA OR "small interfering RNA" OR GalNAc OR "gene silencing" OR "target engagement" OR pharmacokinetics OR therapeutic OR treatment)
**C (Q9)**	TS=("hereditary transthyretin amyloidosis" OR hATTR OR "transthyretin amyloidosis" OR "ocular amyloidosis" OR "vitreous amyloidosis")
**L (Q9)**	TS=("liver transplantation" OR ocular OR vitreous OR retina OR glaucoma)

Exact query history: Q1=(S) AND (H); Q2=(S) AND (O); Q3=(S) AND (H) AND (O); Q4=(S) AND (F) AND ((H) OR (O)); Q5=(S) AND (O) AND (M); Q6=(S) AND ((H) OR (O)) AND (B); Q7=(S) AND ((H) OR (O)) AND (T); Q8=(H) AND (O); Q9=(C) AND (L)

Scopus

Fields: TITLE-ABS-KEY(...). Limits: none. Final search date: 1 May 2026.

Block and modules	Exact database-specific block
**S (Q1-Q7)**	TITLE-ABS-KEY("S100A9" OR "S100A8/A9" OR "S100A8-S100A9" OR calprotectin OR "MRP8/MRP14" OR "MRP8/14" OR "myeloid-related protein 8/14")
**H (Q1,Q3,Q4,Q6-Q8)**	TITLE-ABS-KEY(liver OR hepatic OR MASLD OR "metabolic dysfunction-associated steatotic liver disease" OR MASH OR "metabolic dysfunction-associated steatohepatitis" OR NAFLD OR "nonalcoholic fatty liver disease" OR "non-alcoholic fatty liver disease" OR NASH OR "nonalcoholic steatohepatitis" OR "non-alcoholic steatohepatitis" OR MAFLD OR "metabolic-associated fatty liver disease" OR MetALD OR "metabolic dysfunction and alcohol-associated liver disease" OR "alcohol-related liver disease" OR cirrhosis OR "hepatic fibrosis" OR "drug-induced liver injury" OR DILI OR "ischemia-reperfusion injury" OR "ischaemia-reperfusion injury" OR "viral hepatitis")
**O (Q2-Q8)**	TITLE-ABS-KEY(eye OR ocular OR retina OR retinal OR "diabetic retinopathy" OR "retinal pigment epithelium" OR RPE OR "retinal endothelial" OR "blood-retinal barrier" OR cornea OR corneal OR "ocular surface" OR "dry eye" OR "dry eye disease" OR "lacrimal gland" OR uvea OR uveal OR uveitis OR "acute anterior uveitis" OR "experimental autoimmune uveitis" OR "endotoxin-induced uveitis")
**M (Q5)**	TITLE-ABS-KEY(TLR4 OR "Toll-like receptor 4" OR RAGE OR "receptor for advanced glycation end products" OR "NF-kappa B" OR "NF-κB" OR MAPK OR HIF-1alpha OR HIF-1α OR VEGF OR NLRP3 OR inflammasome OR PP2A OR AMPK OR autophagy OR STAT3 OR Acod1)
**F (Q4)**	TITLE-ABS-KEY(heterodimer OR heterotetramer OR tetramer OR oligomerization OR "calcium binding" OR phosphorylation OR Thr113 OR secretion OR release OR "gasdermin D" OR GSDMD OR "neutrophil extracellular trap" OR NET OR "extracellular vesicle" OR exosome)
**B (Q6)**	TITLE-ABS-KEY(biomarker OR serum OR plasma OR circulating OR tear OR "aqueous humor" OR "aqueous humour" OR vitreous OR "ocular fluid" OR ELISA OR immunoturbidimetry OR "multiplex immunoassay" OR prognostic OR predictive OR monitoring)
**T (Q7)**	TITLE-ABS-KEY(Paquinimod OR Tasquinimod OR "anti-S100A9" OR "S100A9 antibody" OR "neutralizing antibody" OR siRNA OR "small interfering RNA" OR GalNAc OR "gene silencing" OR "target engagement" OR pharmacokinetics OR therapeutic OR treatment)
**C (Q9)**	TITLE-ABS-KEY("hereditary transthyretin amyloidosis" OR hATTR OR "transthyretin amyloidosis" OR "ocular amyloidosis" OR "vitreous amyloidosis")
**L (Q9)**	TITLE-ABS-KEY("liver transplantation" OR ocular OR vitreous OR retina OR glaucoma)

Exact query history: Q1=(S) AND (H); Q2=(S) AND (O); Q3=(S) AND (H) AND (O); Q4=(S) AND (F) AND ((H) OR (O)); Q5=(S) AND (O) AND (M); Q6=(S) AND ((H) OR (O)) AND (B); Q7=(S) AND ((H) OR (O)) AND (T); Q8=(H) AND (O); Q9=(C) AND (L)

Table S1E. Duplicate handling and structured summary of evidence selection and claim-level appraisal

Domain	Procedure
**Duplicate handling**	Duplicate references retrieved across databases or citation searches were identified during reference management using DOI, PMID, title, first author, and publication year, and were retained once. Because the searches were iterative and claim-directed, no fixed deduplicated screening library was created.
**Formal screening count**	No formal screening log, PRISMA-style flow diagram, approximate category-specific study count, or formally delimited set of included studies was maintained.
**Review type**	Structured narrative and hypothesis-generating review informed by SANRA [38].
**Retrieval approach**	Iterative, targeted, and claim-directed rather than exhaustive. Search modules were rerun or refined when a claim required source-, matrix-, disease-, model-, molecular-form-, intervention-, or safety-specific support.
**Evidence considered**	Human clinical studies, ocular-fluid studies, animal models, cell-culture studies, molecular or structural studies, assay studies, and pharmacological or safety studies relevant to the stated claims.
**Primary evidence preference**	Primary studies were prioritized. Reviews were used mainly for contextual framing, terminology, and identification of primary literature.
**Cross-tissue evidence**	Non-hepatic or non-ocular studies were retained only when they directly informed molecular organization, release, receptor interaction, assay interpretation, pharmacology, or safety. They were classified as limited or indirect support, not as direct ocular or hepato-ocular evidence.
**Not used as primary support**	Unrelated S100-family proteins; computational-only predictions without experimental validation; studies lacking an identifiable analyte or biological context; and remote tissue analogies without direct relevance to the claim.
**Claim-support categories**	Context-specific direct support (CD), limited or indirect support (LI), and hypothesis only (HO). These categories describe claim-level directness and coherence, not study quality, formal risk of bias, quantitative certainty, or GRADE certainty.
**Appraisers**	Peng Wang and Yamei Li independently appraised the major claims and the studies selected to support them.
**Disagreement resolution**	Disagreements were resolved by discussion and re-examination of primary studies; unresolved cases were adjudicated by Qiuxian Peng.
**Predefined appraisal considerations**	Claim directness; molecular-species, assay, source, matrix, and compartment specificity; design relevance to association, temporality, transport, target engagement, or intervention; consistency; and threats to inference, including confounding, reverse causation, non-selective pharmacology, incomplete target engagement, and cross-context extrapolation.

Domain	Procedure
**Scope of appraisal**	The appraisal was applied to the major claims and the studies selected to support them, not to every record retrieved during iterative searches. Table 1 summarizes the claim domains, claim-support categories, and concise interpretations; Supplementary Table S2 provides the non-exhaustive claim–study mapping.
**Quantitative synthesis**	Not undertaken because of heterogeneity in disease definitions, molecular species, matrices, assays, experimental systems, interventions, and outcomes.
**Formal risk-of-bias or GRADE assessment**	Not undertaken because the article was not designed as a systematic review, scoping review, or meta-analysis.
