# Supplementary material for: S100A9 as a Candidate Molecular Bridge in Hepato–Ocular Crosstalk
Source: Biology (Basel). 2026 Aug 5;15(15):1306. doi: 10.3390/biology15151306 (PMC13464601; doi:10.3390/biology15151306)
Supplement: Supplementary file 1 [file biology-15-01306-s001.zip › Supplementary Table S2 Study-Level Evidence Matrix Underpinning the Claim-Level Appraisal in Table 1.pdf]

Supplementary Table S2

Supplementary Table S2. Study-level evidence matrix underpinning the claim-level appraisal in Table 1.

The main-text Table 1 contains only the claim domain, claim-support category, and concise interpretation. This supplementary table provides the detailed evidence types, reference numbers, disease or experimental contexts, biological compartments, molecular species or interventions, study-specific contributions, and principal limitations. Reference numbers correspond to the final main-manuscript reference list (1–76). Primary studies are mapped according to the claims they inform; reviews are not treated as independent primary evidence. Non-hepatic, non-ocular, and cross-disease studies are explicitly classified as indirect biological context. The matrix is representative rather than exhaustive.

Table S2A. Claim domains and main-table appraisal categories

Claim code	Claim domain	Main Table 1 category
C1	Hepatic expression	CD
C2	Release and potential systemic availability	LI
C3	Hepatic-to-ocular transport or systemic contribution	HO
C4	Ocular receptor competence and TLR4-associated responsiveness	CD/LI
C5	Retinal evidence	CD/LI
C6	Ocular-surface and dry-eye evidence	CD/LI
C7	Uveitis evidence	CD/LI
C8	Biomarker and translational implications	LI/HO

CD, context-specific direct support; LI, limited or indirect support; HO, hypothesis only. Mixed categories indicate that different components of the same claim have different levels of support.

Table S2B. Detailed claim–study matrix

Claim	Ref.	Evidence type	Disease/model and compartment	Molecular species or intervention	Contribution and principal limitation	Appraisal role
C1	[23]	HC/tissue	Human MASLD/MASH; paired liver and blood; hepatic myeloid populations	S100A9-high hepatic myeloid populations	Identifies TREM2+S100A9+ macrophages and other S100-high immune populations. Direct for hepatic cellular presence; does not demonstrate extracellular release or quantify hepatic contribution to circulating material.	CD
C1	[19]	AM/CC	Experimental hepatic fibrosis; liver and hepatic macrophages	S100A8/A9; TLR4-associated macrophage signaling	Supports local S100A8/A9-associated macrophage activation. Does not quantify systemic release or establish ocular effects.	CD
C1	[21]	AM/CC	Fatty-liver injury and fibrosis; neutrophils, stellate cells, and liver	Neutrophil-secreted S100A8/A9	Supports neutrophil-derived S100A8/A9 and myofibroblast migration. Model-specific; no ocular outcome or source-resolved systemic measurement.	CD
C1	[22]	AM	Obesity-associated MASLD; adipose tissue and hepatic infiltrating macrophages	S100A8-positive macrophages	Supports hepatic infiltration of S100A8-positive macrophages. The analyte is S100A8, not isolated S100A9 or a defined S100A8/A9 complex.	CD
C1	[24]	AM/CC	Steatosis-to-MASH progression; adipose tissue and liver	S100A8/A9-associated adipose-liver signaling	Supports disease amplification, but the initiating source is substantially extrahepatic and the ocular compartment was not examined.	LI
C1	[20]	AM genetic	Sepsis-induced acute liver injury; liver and systemic metabolism	S100A9 deficiency	Deficiency reduced hepatic injury. The systemic sepsis model does not permit liver-specific source attribution.	CD
C1	[25]	AM intervention	CCl4-induced acute liver injury; liver tissue	Hepatic S100A8 and S100A9; non-selective herbal intervention	Shows treatment-associated hepatic expression changes. The intervention does not establish S100A9-specific causality.	LI
C1	[26]	AM/CC	Fatty-liver ischemia-reperfusion; hepatic macrophages and liver	S100A9-TLR2-NLRP3-associated signaling	Supports a model-specific local mechanism. Does not establish systemic availability or ocular effects.	CD
C1	[44]	AM/CC	Experimental liver fibrosis; hepatic macrophages	S100A8-NLRP3-dependent macrophage pyroptosis	Supports S100A8-dependent macrophage pyroptosis, not isolated S100A9.	CD
C1	[27]	AM/CC	Echinococcus-associated liver fibrosis; macrophages, stellate cells, and liver	Macrophage-derived S100A9; SPARC	Supports local profibrotic S100A9 activity in an infectious model. Generalizability is limited.	CD
C1/C3/C7	[57]	Integrated AM	Acute D-galactosamine/LPS liver injury with ocular inflammation; liver and ocular tissues	S100A9-associated changes; PP2A-AMPK; polyphenol intervention	Concurrent hepatic and ocular changes were measured. Systemic LPS may activate both organs in parallel; no source tracing, biodistribution, or source-selective perturbation was performed.	C1: CD for local hepatic change; C3: LI/HO for systemic linkage; C7: LI for integrated interpretation
C2	[30]	CC mechanistic	Activated neutrophils; extracellular medium	Thr113-phosphorylated S100A9	Supports neutrophil release of phosphorylated S100A9. This is not hepatic- or ocular-source evidence.	LI
C2	[40]	Mechanistic, non-hepatic	Familial Mediterranean fever; neutrophils	S100A8/A9; gasdermin-D-pore-associated secretion	Supports regulated S100A8/A9 secretion in systemic autoinflammation. Quantitative relevance to liver disease is unknown.	LI

Claim	Ref.	Evidence type	Disease/model and compartment	Molecular species or intervention	Contribution and principal limitation	Appraisal role
C2	[43]	Mechanistic, non-hepatic	Neutrophil activation; neutrophils	E-selectin/NLRP3-associated S100A8/A9 release through transient gasdermin-D pores	Supports a defined neutrophil release mechanism. Hepatic and ocular relevance was not tested.	LI
C2	[41]	CC mechanistic	NET formation; neutrophils and NET-associated material	S100A8/A9-associated material	Supports association with NET release and neutrophil activation. Not liver-specific.	LI
C2	[31]	Human/AM cross-tissue	Septic shock and acute lung injury; circulating EVs and lung	EV-bound S100A8/A9	Demonstrates a circulating EV-associated form. Hepatic origin and ocular delivery were not tested.	LI
C2	[42]	Human/CC cross-tissue	Burn injury and cardiomyocyte pyroptosis; serum exosomes and cardiomyocytes	Serum-exosomal S100A9	Supports possible distant exosomal activity. The burn/myocardial context does not establish a liver-eye mechanism.	LI
C3	[46]	HC	Type 2 diabetes with DR; blood	S100A8 and S100A9 measured separately	Association with DR severity does not demonstrate heterocomplex formation, hepatic origin, ocular entry, or mediation.	LI for systemic association; HO for hepatic origin, ocular entry, or mediation
C3/C5	[10]	OF/AM	Human DR vitreous and diabetic rat retina	S100A9/TLR4-associated proteins; Paquinimod	Supports ocular-compartment abundance and experimental retinal response. Vitreous origin and the site of action of systemic treatment remain unresolved.	C3: LI for ocular-compartment exposure and HO for hepatic transport; C5: LI human / CD animal
C3/C6	[11]	AM/CC	Lacrimal-gland-excision DED; corneal epithelium	S100A9-TLR4-associated signaling; Paquinimod/TAK-242	Supports a local ocular-surface mechanism, not liver-derived exposure.	C3: HO for hepatic contribution; C6: CD for local model activity
C3/C6	[12]	Human cellular/AM	Sjögren-related DED; PBMCs, lacrimal gland, and dendritic cells	S100A8/A9 complex-Acod1/STAT3; Paquinimod	Supports S100A8/A9-complex-associated immune amplification. Does not establish hepatic isolated S100A9.	C3: HO for hepatic isolated S100A9; C6: CD for local S100A8/A9-associated activity
C3/C7	[13]	AM intervention	EIU; anterior ocular tissues and aqueous humor	S100A9-positive cells; anti-S100A9 antibody	Supports local infiltrating cells and partial target perturbation. No hepatic attribution.	C3: HO for hepatic contribution; C7: CD for local EIU involvement
C3/C7	[54]	HC	Active acute anterior uveitis; plasma	Plasma S100A8/A9 immunoreactivity	Tracks inflammatory activity and corticosteroid-associated improvement. Exact oligomeric state, hepatic origin, and ocular transport were not tested.	C3: LI for systemic association and HO for hepatic transport; C7: LI
C3/C7	[56]	HC/OF	JIA-associated and idiopathic anterior uveitis; serum and aqueous humor	Serum S100A8/A9 and S100A12; aqueous-humor S100A8/A9 immunoreactivity	Supports selected systemic and ocular-fluid associations. Exact oligomeric state, source, transport direction, and prospective utility remain unresolved.	C3: LI for parallel systemic/ocular-fluid association and HO for transport direction; C7: LI
C3	No direct study	Hypothesis	Proposed liver-specific ocular delivery; liver, blood, and ocular compartments	Defined liver-derived S100A9-related species	No study connects hepatic production, source-resolved systemic release, ocular biodistribution, target engagement, and ocular effect.	HO
C4	[33]	AM genetic	Experimental diabetic retina; retinal endothelial and Müller cells	Cell-specific TLR4 deletion	Demonstrates cell-specific retinal TLR4 function. S100A9 was not established as the relevant ligand.	CD for TLR4 competence
C4/C5	[10]	OF/AM	Human DR vitreous and diabetic rat retina	S100A9/TLR4-associated proteins; Paquinimod	Supports selected retinal S100A9-TLR4-associated responses. Direct in vivo ligand-receptor binding or receptor occupancy was not tested.	C4: CD local / LI engagement; C5: LI human / CD animal
C4/C6	[11]	AM/CC	Lacrimal-gland-excision DED; corneal epithelium	S100A9-TLR4-associated signaling; Paquinimod/TAK-242	Supports model-specific ocular-surface activity. Direct ligand-receptor binding was not examined.	C4: CD local / LI engagement; C6: CD
C4	[34]	CC, indirect ocular context	Hyperglycemia-exposed retinal Müller glia	RAGE-dependent inflammatory signaling; S100A9 not tested	Supports ocular RAGE competence under hyperglycemic conditions, but does not demonstrate S100A9 as the ligand or establish hepato-ocular signaling.	LI
C4	[35]	CC/experimental, indirect ocular context	Diabetic retinal endothelial matrix-stiffening model	RAGE-mediated endothelial activation; S100A9 not tested	Supports context-dependent retinal endothelial RAGE activity. It is not direct evidence of ocular S100A9-RAGE engagement.	LI
C4	[36]	CC/AM, indirect ocular context	Diabetic retinal pigment epithelial cells and retina	HMGB1-related signaling; S100A9 not tested	Provides indirect context for an alternative RAGE-associated ligand in retinal disease and does not support S100A9-specific receptor occupancy.	LI
C5	[46]	HC	Type 2 diabetes with DR; blood	Circulating S100A8 and S100A9 measured separately	Cross-sectional and source-unresolved; does not establish S100A8/A9-complex activity, temporal prediction, or hepatic mediation.	LI
C5	[10]	OF/AM	Human DR vitreous and diabetic rat retina	S100A9/TLR4-associated proteins; Paquinimod	Supports vitreous association and animal-model pathway modulation. Hepatic mediation and S100A9-specific receptor engagement were not established.	LI human; CD animal
C5	[48]	CC	High-glucose-treated human retinal endothelial cells	S100A9 expression; Tasquinimod	Supports retinal endothelial responses in cell culture. Tasquinimod is pleiotropic; in vivo efficacy and S100A9-specific target engagement were not established.	CD cell; LI specificity
C6	[8]	HC	Selected liver diseases and DED; ocular surface and tears	Ocular phenotype; S100A9-related species not measured	Supports disease-specific clinical associations only. A shared S100A9-related mechanism was not assessed.	LI
C6	[51]	HC	Primary biliary cholangitis with seasonal allergic conjunctivitis; ocular surface	Ocular phenotype; S100A9-related species not measured	Coexisting seasonal allergic conjunctivitis limits attribution to primary biliary cholangitis.	LI

Claim	Ref.	Evidence type	Disease/model and compartment	Molecular species or intervention	Contribution and principal limitation	Appraisal role
C6	[11]	AM/CC	Lacrimal-gland-excision DED; corneal epithelium	S100A9-TLR4-associated signaling; Paquinimod/TAK-242	Supports local activity in an animal model. Direct receptor engagement was not measured.	CD
C6	[12]	Human cellular/AM	Sjögren-related DED; PBMCs, lacrimal gland, and dendritic cells	S100A8/A9 complex-Acod1/STAT3; Paquinimod	Supports a distinct S100A8/A9-complex-associated immune mechanism; not isolated S100A9.	CD
C7	[54]	HC	Active acute anterior uveitis; plasma	S100A8/A9 immunoreactivity	Elevated in active disease and declined with corticosteroid-associated improvement. Exact oligomeric state, source, and ocular transport were not assessed.	LI
C7	[55]	HC	Idiopathic acute anterior uveitis; serum	Calprotectin immunoreactivity; TLR4/MyD88 measurements	Serum calprotectin was increased and correlated with clinical activity and macular thickness. The assay does not establish a specific oligomeric form, ocular receptor activation, or calprotectin as the initiating ligand.	LI
C7	[56]	HC/OF	JIA-associated and idiopathic anterior uveitis; serum and aqueous humor	Serum S100A8/A9 and S100A12; aqueous-humor S100A8/A9 immunoreactivity	Supports selected systemic and ocular-fluid associations. Exact oligomeric state, source, transport direction, and prospective utility remain unresolved.	LI
C7	[13]	AM intervention	EIU; iris-ciliary body, cornea, and aqueous humor	S100A9-positive cells; anti-S100A9 antibody	Supports local myeloid-cell infiltration and partial antibody-associated reduction of inflammation. Model-specific.	CD
C7	[57]	Integrated AM	Acute D-galactosamine/LPS liver injury with ocular inflammation; liver and ocular tissues	S100A9-associated changes; PP2A-AMPK; polyphenol intervention	Supports concurrent changes in both organs. Systemic LPS may activate them in parallel; no source tracing was performed.	LI
C7	[58]	AM proteomics	EAU and primed mycobacterial uveitis; aqueous humor and vitreous	Calprotectin immunoreactivity; S100A8 and S100A9 abundance	Supports model-dependent ocular-fluid abundance rather than a universal uveitis pathway. Exact molecular forms were not resolved.	LI
C8	[32]	Analytical	Circulating calprotectin measurement; serum versus plasma	S100A8/A9 immunoreactivity; oligomeric state unresolved	Demonstrates matrix and pre-analytical effects; does not validate a hepato-ocular biomarker.	CD assay interpretation
C8	[10]	OF/AM intervention	Human DR vitreous and diabetic rat retina	Paquinimod; S100A9/TLR4-associated proteins	Supports preclinical retinal pathway modulation. Molecular-form specificity, ocular target engagement, and clinical efficacy remain unresolved.	LI
C8	[11]	AM/CC intervention	Lacrimal-gland-excision DED	Paquinimod; TAK-242	Supports preclinical ocular-surface pathway modulation. No ophthalmic clinical efficacy has been established.	LI
C8	[12]	Human cellular/AM intervention	Sjögren-related DED	Paquinimod; S100A8/A9 complex-Acod1/STAT3	Supports preclinical modulation of an S100A8/A9-associated immune pathway. Isolated S100A9 specificity is not established.	LI
C8	[13]	AM intervention	EIU	Anti-S100A9 antibody	Supports partial preclinical reduction of acute ocular inflammation. Route, molecular-form specificity, and clinical relevance remain unresolved.	LI
C8	[48]	CC intervention	High-glucose-treated human retinal endothelial cells	Tasquinimod	Supports cell-culture pathway modulation. Pleiotropic pharmacology limits S100A9-specific inference.	LI
C8	[61]	Human interventional, non-ocular	Systemic lupus erythematosus; systemic exposure	Paquinimod	Provides non-ophthalmic human pharmacokinetic and tolerability evidence only; ocular exposure, ophthalmic dosing, and hepato-ocular efficacy were not evaluated.	LI
C8	[62]	Platform	Hepatocyte-directed RNAi; hepatocytes	GalNAc-siRNA	Demonstrates hepatocyte-enriched delivery as a platform, not an S100A9-directed product. Hepatocyte targeting may not address predominantly myeloid hepatic sources.	HO translational
C8	No direct study	Hypothesis	Clinical hepato-ocular application; clinical care	S100A9-related biomarker or intervention	No analyte has established incremental clinical utility, and no source-selective S100A9 intervention has demonstrated ocular benefit.	HO

Abbreviations: AAU, acute anterior uveitis; AM, animal model; CC, cell culture; CD, context-specific direct support; DED, dry eye disease; DR, diabetic retinopathy; EAU, experimental autoimmune uveitis; EIU, endotoxin-induced uveitis; EV, extracellular vesicle; HC, human clinical evidence; HO, hypothesis only; JIA-U, juvenile idiopathic arthritis-associated uveitis; LI, limited or indirect support; LPS, lipopolysaccharide; NET, neutrophil extracellular trap; OF, ocular-fluid evidence; PBMC, peripheral blood mononuclear cell; RNAi, RNA interference.

Interpretive note: Appraisal-role labels indicate how an individual study contributes to the specified claim; for multi-claim rows, the role is stated separately by claim. These labels do not represent study quality, formal risk of bias, quantitative certainty, or GRADE ratings. No formal PRISMA-style screening count was maintained. Where studies reported S100A8/A9 or calprotectin immunoreactivity, the exact heterodimeric versus higher-order oligomeric state was considered unresolved unless directly characterized.
