# Supplementary material for: S100A9 as a Candidate Molecular Bridge in Hepato–Ocular Crosstalk
Source: Biology (Basel). 2026 Aug 5;15(15):1306. doi: 10.3390/biology15151306 (PMC13464601; doi:10.3390/biology15151306)
Supplement: Supplementary file 1 [file biology-15-01306-s001.zip › Supplementary Table S3 Representative Evidence and Developmental Boundaries of Experimental S100A9-Related Intervention Concepts.pdf]

Supplementary Table S3

Supplementary Table S3. Representative Evidence and Developmental Boundaries of Experimental S100A9-Related Intervention Concepts

This supplementary table contains the representative evidence removed from main-text Table 2 and provides the detailed study-level basis for each intervention concept. References correspond to the final main-manuscript sequence (1–76). Non-ocular mechanistic, platform, and safety studies are classified as indirect context rather than evidence of hepato–ocular efficacy.

Table S3A. Mapping of the Main Table 2 Intervention Concepts to Representative Evidence

Intervention concept	Representative evidence moved from Table 2	Refs.	Evidence stage
Paquinimod-mediated systemic modulation	S100A9-binding evidence; systemic Paquinimod in experimental DR, lacrimal-excision DED, and Sjögren-related DED; experimental liver fibrosis; and non-ophthalmic human pharmacokinetic and tolerability data.	[10–12,59–61]	ME; OAM; HAM; NHC
Tasquinimod-mediated pathway modulation	High-glucose-treated human retinal endothelial cells, with HDAC4-related and broader myeloid actions characterized in non-ocular models.	[48–50,64]	OCC; IM
Experimental anti-S100A9 antibody intervention	Partial reduction of inflammation and cellular infiltration in endotoxin-induced uveitis, supplemented by indirect antibody-development requirements concerning Fc function and characterization.	[13,69]	OAM; IS
Hepatic or myeloid source-directed modulation	Hepatocyte-enriched GalNAc–siRNA delivery as a platform concept and mapping of hepatic myeloid populations relevant to S100A9-related expression.	[23,62,63]	PC
Local ocular S100A9-directed intervention	No directly developed topical, periocular, or intraocular S100A9-directed formulation was identified. Basic and non-ocular studies provide only indirect safety context for host defense and tissue repair.	No direct study; indirect context [15,18,65–68]	HY; IS

Table S3B. Detailed Study-Level Evidence and Developmental Boundaries

Main Table 2 concept	Specific evidence item	Ref.	Evidence setting	Target or route	Contribution and principal boundary	Stage
Paquinimod-mediated systemic modulation	Paquinimod: molecular interaction	[59]	Non-ocular mechanistic study	Quinoline-3-carboxamide binding to S100A9-associated extracellular signaling	Supports pharmacological tractability. The study does not resolve all S100A9-related molecular forms or distinguish hepatic, circulating, and ocular sources.	ME
Paquinimod-mediated systemic modulation	Paquinimod: experimental DR	[10]	Human ocular-fluid association and diabetic-rat model	Systemic administration; S100A9/TLR4-associated pathway	Reduced selected retinal abnormalities together with pathway-associated protein changes. The source of the ocular signal and direct in vivo ocular target engagement were not established.	OAM
Paquinimod-mediated systemic modulation	Paquinimod: lacrimal-excision DED	[11]	Ocular animal and cell models	Systemic Paquinimod with TLR4-pathway comparison	Improved selected ocular-surface, inflammatory, and autophagy-related outcomes. Evidence is limited to one model, without a local formulation or ocular pharmacokinetic data.	OAM
Paquinimod-mediated systemic modulation	Paquinimod: Sjögren-related DED	[12]	Human cellular and animal evidence	Systemic modulation of S100A8/A9–Acod1/STAT3-associated activity	Improved selected manifestations in non-obese diabetic mice. The relevant analyte was S100A8/A9 rather than isolated S100A9, and hepatic origin was not examined.	OAM
Paquinimod-mediated systemic modulation	Paquinimod: liver fibrosis	[60]	Hepatic animal model	Oral systemic immunomodulation	Reversed established fibrosis in a mouse model. The intervention was not hepatic-source-selective, and no ocular outcome or coordinated hepato–ocular effect was assessed.	HAM
Paquinimod-mediated systemic modulation	Paquinimod: human exposure	[61]	Non-ophthalmic clinical study	Repeated systemic dosing	Provides pharmacokinetic and tolerability information only. Ocular exposure, ophthalmic dosing, target engagement, and hepato–ocular efficacy were not evaluated.	NHC

Main Table 2 concept	Specific evidence item	Ref.	Evidence setting	Target or route	Contribution and principal boundary	Stage
Tasquinimod-mediated pathway modulation	Tasquinimod: retinal endothelial model	[48]	Ocular cell culture	High-glucose-treated human retinal endothelial cells	Reduced proliferation, migration, tube formation, and selected signaling responses. S100A9-specific engagement, in vivo ophthalmic efficacy, and ocular exposure were not demonstrated.	OCC
Tasquinimod-mediated pathway modulation	Tasquinimod: HDAC4 activity	[49]	Non-ocular mechanistic study	Allosteric HDAC4 modulation	Demonstrates a molecular action beyond an S100A9-only mechanism and limits attribution of retinal endothelial effects specifically to S100A9.	IM
Tasquinimod-mediated pathway modulation	Tasquinimod: broader myeloid modulation	[50,64]	Non-ocular cancer and mechanistic models	Systemic modulation of suppressive myeloid populations	Demonstrates pleiotropic myeloid and antitumor effects. These studies do not establish an ocular mechanism, ophthalmic target engagement, or ocular safety.	IM
Experimental anti-S100A9 antibody intervention	Anti-S100A9 antibody: EIU	[13]	Ocular animal model	Experimental antibody intervention against extracellular S100A9-associated activity	Partially reduced acute ocular inflammation and cellular infiltration. Molecular form, epitope, ocular exposure, Fc-mediated effects, and chronic-disease relevance remain unresolved.	OAM
Hepatic or myeloid source-directed modulation	Hepatocyte-directed GalNAc–siRNA	[62]	Delivery-platform evidence	ASGPR-mediated hepatocyte-enriched RNA interference	Validates hepatocyte-enriched delivery as a platform, not an S100A9-directed product. It may not address Kupffer cells, recruited macrophages, or neutrophils, which are prominent hepatic S100A9-related populations.	PC
Hepatic or myeloid source-directed modulation	Hepatic or systemic myeloid source modulation	[23,63]	Human cellular mapping and liver immunology	Kupffer cells, recruited macrophages, and other hepatic myeloid populations	Identifies biologically relevant source populations but does not provide an organ-selective intervention. Broad myeloid modulation would also affect circulating and extrahepatic cells.	PC
Cross-cutting development requirement	Molecular-form specificity requirement	[15,28,29,31]	Structural, biochemical, and cross-tissue evidence	Isolated S100A9, S100A8/A9 heterocomplexes, higher-order assemblies, and EV-associated material	Shows that S100A9-related analytes are not interchangeable. Any intervention must define the targeted molecular species and demonstrate analyte-specific target modulation.	IM
Cross-cutting development requirement	Antibody Fc-function requirements	[69]	Non-ocular antibody-development context	Fc-mediated effector functions and antibody characterization	Provides general antibody-development requirements only. Fc function, immune-complex behavior, and ocular consequences have not been characterized for an anti-S100A9 ophthalmic candidate.	IS
Cross-cutting development requirement	Systemic host-defense and repair constraints	[15,18,65–68]	Basic and non-ocular safety context	Systemic S100A9 or S100A8/A9 modulation	Supports context-dependent roles in host defense, leukocyte recruitment, regeneration, and immunity. Direct safety consequences in hepato–ocular disease remain unknown, and advanced liver disease may increase infection vulnerability.	IS
Local ocular S100A9-directed intervention	Topical ocular S100A9 intervention	No direct study	Hypothesis	Proposed topical delivery of a defined S100A9-related target	No S100A9-directed topical formulation, ocular pharmacokinetic study, or target-modulation assay was identified. Target species, penetration, dose response, reversibility, host defense, and repair effects remain undefined.	HY
Local ocular S100A9-directed intervention	Periocular or intraocular S100A9 intervention	No direct study	Hypothesis	Proposed periocular or intraocular small-molecule, antibody, or nucleic-acid modality	No S100A9-specific ocular biodistribution, target-engagement, toxicity, durability, or formulation data were identified.	HY

Abbreviations: ASGPR, asialoglycoprotein receptor; DED, dry eye disease; DR, diabetic retinopathy; EIU, endotoxin-induced uveitis; EV, extracellular vesicle; HAM, hepatic animal-model evidence; HY, hypothesis only; IM, indirect mechanistic context; IS, indirect safety context; ME, mechanistic evidence; NHC, non-ophthalmic human clinical exposure; OAM, ocular animal-model evidence; OCC, ocular cell-culture evidence; PC, platform concept; TLR4, Toll-like receptor 4. Evidence-stage labels describe evidence source and developmental maturity rather than treatment efficacy, study quality, formal risk of bias, quantitative certainty, or GRADE ratings. No S100A9-directed topical, periocular, or intraocular formulation was identified in the reviewed literature.
