# Supplementary material for: S100A9 as a Candidate Molecular Bridge in Hepato–Ocular Crosstalk
Source: Biology (Basel). 2026 Aug 5;15(15):1306. doi: 10.3390/biology15151306 (PMC13464601; doi:10.3390/biology15151306)
Supplement: Supplementary file 1 [file biology-15-01306-s001.zip › Supplementary Text S1 Additional Information on Review Methodology.pdf]

Supplementary Text S1

Additional Information on Review Methodology

This supplementary text provides the detailed evidence-identification and claim-level appraisal procedures referred to in Section 2 of the main manuscript.

The searches were iterative and claim-directed. Human studies, ocular-fluid investigations, animal and cell models, and relevant molecular or structural studies were considered. Non-hepatic or non-ocular studies were used only to inform molecular organization, release mechanisms, receptor interactions, assay interpretation, pharmacology, or safety and were explicitly treated as indirect biological context. Studies with poorly defined molecular species or remote tissue analogies were not treated as primary support for hepato–ocular claims.

Peng Wang and Yamei Li independently appraised the major claims and the studies selected to support them. The appraisal was applied to claim-supporting evidence rather than to every record retrieved during the iterative searches; consequently, no formally delimited set of "included studies" was established. The appraisal addressed claim directness; molecular-species, assay, source, matrix, and compartment specificity; design relevance; consistency; and major threats to inference, including confounding, reverse causation, non-selective pharmacology, incomplete target engagement, and cross-context extrapolation. Primary studies were prioritized, with reviews used mainly for contextual framing and identification of primary literature.

Claims were assigned to three claim-support categories—"context-specific direct support" (CD), "limited or indirect support" (LI), and "hypothesis only" (HO)—rather than evidence-certainty grades. CD required direct examination of the specific claim component in the relevant disease or experimental compartment, sufficiently defined molecular species or analytes, and findings that were broadly concordant within that context. LI included cross-sectional, model-specific, unreplicated, incompletely characterized, substantially confounded, pharmacologically non-selective, or partial-pathway evidence. HO denoted biological plausibility or integration across separate systems without direct testing. Molecular-species ambiguity, unresolved source attribution, or material inconsistency precluded classification as CD.

Disagreements were resolved through discussion and re-examination of the primary studies, with unresolved cases adjudicated by Qiuxian Peng. Table 1 summarizes the claim domains, claim-support categories, and concise interpretations, whereas Supplementary Table S2 provides detailed study types, reference numbers, disease or experimental contexts, molecular species, and claim-specific limitations. No formal screening log or PRISMA-style study-flow count was maintained.
